# *MtGSTF7*, a TT19-like GST gene, is essential for accumulation of anthocyanins, but not proanthocyanins in *Medicago truncatula*

**DOI:** 10.1093/jxb/erac112

**Published:** 2022-03-16

**Authors:** Ruoruo Wang, Nan Lu, Chenggang Liu, Richard A Dixon, Qing Wu, Yawen Mao, Yating Yang, Xiaoling Zheng, Liangliang He, Baolin Zhao, Fan Zhang, Shengchao Yang, Haitao Chen, Ji Hyung Jun, Ying Li, Changning Liu, Yu Liu, Jianghua Chen

**Affiliations:** CAS Key Laboratory of Tropical Plant Resources and Sustainable Use, CAS Center for Excellence for Molecular Plant Science, Xishuangbanna Tropical Botanical Garden, Chinese Academy of Sciences, Kunming, Yunnan 650223, China; University of Chinese Academy of Sciences, Beijing 100049, China; Yunnan Key Laboratory of Plant Reproductive Adaptation and Evolutionary Ecology and Institute of Biodiversity, School of Ecology and Environmental Science, Yunnan University, Kunming, Yunnan 650500, China; BioDiscovery Institute and Department of Biological Sciences, University of North Texas, Denton, TX 76203, USA; BioDiscovery Institute and Department of Biological Sciences, University of North Texas, Denton, TX 76203, USA; BioDiscovery Institute and Department of Biological Sciences, University of North Texas, Denton, TX 76203, USA; CAS Key Laboratory of Tropical Plant Resources and Sustainable Use, CAS Center for Excellence for Molecular Plant Science, Xishuangbanna Tropical Botanical Garden, Chinese Academy of Sciences, Kunming, Yunnan 650223, China; University of Chinese Academy of Sciences, Beijing 100049, China; CAS Key Laboratory of Tropical Plant Resources and Sustainable Use, CAS Center for Excellence for Molecular Plant Science, Xishuangbanna Tropical Botanical Garden, Chinese Academy of Sciences, Kunming, Yunnan 650223, China; University of Chinese Academy of Sciences, Beijing 100049, China; CAS Key Laboratory of Tropical Plant Resources and Sustainable Use, CAS Center for Excellence for Molecular Plant Science, Xishuangbanna Tropical Botanical Garden, Chinese Academy of Sciences, Kunming, Yunnan 650223, China; School of Life Science, University of Science and Technology of China, Hefei, Anhui 230026, China; CAS Key Laboratory of Tropical Plant Resources and Sustainable Use, CAS Center for Excellence for Molecular Plant Science, Xishuangbanna Tropical Botanical Garden, Chinese Academy of Sciences, Kunming, Yunnan 650223, China; University of Chinese Academy of Sciences, Beijing 100049, China; CAS Key Laboratory of Tropical Plant Resources and Sustainable Use, CAS Center for Excellence for Molecular Plant Science, Xishuangbanna Tropical Botanical Garden, Chinese Academy of Sciences, Kunming, Yunnan 650223, China; CAS Key Laboratory of Tropical Plant Resources and Sustainable Use, CAS Center for Excellence for Molecular Plant Science, Xishuangbanna Tropical Botanical Garden, Chinese Academy of Sciences, Kunming, Yunnan 650223, China; CAS Key Laboratory of Tropical Plant Resources and Sustainable Use, CAS Center for Excellence for Molecular Plant Science, Xishuangbanna Tropical Botanical Garden, Chinese Academy of Sciences, Kunming, Yunnan 650223, China; National and Local Joint Engineering Research Center on Germplasm Innovation and Utilization of Chinese Medicinal Materials in Southwest China, Yunnan Agricultural University, Kunming, Yunnan 650201, China; Sanjie Institute of Forage, Yangling, Shaanxi 712100, China; BioDiscovery Institute and Department of Biological Sciences, University of North Texas, Denton, TX 76203, USA; BioDiscovery Institute and Department of Biological Sciences, University of North Texas, Denton, TX 76203, USA; CAS Key Laboratory of Tropical Plant Resources and Sustainable Use, CAS Center for Excellence for Molecular Plant Science, Xishuangbanna Tropical Botanical Garden, Chinese Academy of Sciences, Kunming, Yunnan 650223, China; CAS Key Laboratory of Tropical Plant Resources and Sustainable Use, CAS Center for Excellence for Molecular Plant Science, Xishuangbanna Tropical Botanical Garden, Chinese Academy of Sciences, Kunming, Yunnan 650223, China; CAS Key Laboratory of Tropical Plant Resources and Sustainable Use, CAS Center for Excellence for Molecular Plant Science, Xishuangbanna Tropical Botanical Garden, Chinese Academy of Sciences, Kunming, Yunnan 650223, China; Yunnan Key Laboratory of Plant Reproductive Adaptation and Evolutionary Ecology and Institute of Biodiversity, School of Ecology and Environmental Science, Yunnan University, Kunming, Yunnan 650500, China; School of Life Science, University of Science and Technology of China, Hefei, Anhui 230026, China; New Zealand

**Keywords:** Anthocyanin, glutathione-*S*-transferase, LAP1, *Medicago truncatula*, MtGSTF7, proanthocyanidin

## Abstract

Anthocyanins and proanthocyanins (PAs) are two end products of the flavonoid biosynthesis pathway. They are believed to be synthesized in the endoplasmic reticulum and then sequestered into the vacuole. In *Arabidopsis thaliana*, TRANSPARENT TESTA 19 (TT19) is necessary for both anthocyanin and PA accumulation. Here, we found that MtGSTF7, a homolog of AtTT19, is essential for anthocyanin accumulation but not required for PA accumulation in *Medicago truncatula*. *MtGSTF7* was induced by the anthocyanin regulator LEGUME ANTHOCYANIN PRODUCTION 1 (LAP1), and its tissue expression pattern correlated with anthocyanin deposition in *M. truncatula*. *Tnt1*-insertional mutants of *MtGSTF7* lost anthocyanin accumulation in vegetative organs, and introducing a genomic fragment of *MtGSTF7* could complement the mutant phenotypes. Additionally, the accumulation of anthocyanins induced by LAP1 was significantly reduced in *mtgstf7* mutants. Yeast-one-hybridization and dual-luciferase reporter assays revealed that LAP1 could bind to the *MtGSTF7* promoter to activate its expression. Ectopic expression of *MtGSTF7* in *tt19* mutants could rescue their anthocyanin deficiency, but not their PA defect. Furthermore, PA accumulation was not affected in the *mtgstf7* mutants. Taken together, our results show that the mechanism of anthocyanin and PA accumulation in *M. truncatula* is different from that in *A. thaliana,* and provide a new target gene for engineering anthocyanins in plants.

## Teaser

*MtGSTF7*, a TT19-like *GST* gene, is activated by the transcription factor LAP1 to determine the accumulation of anthocyanins, but not proanthocyanins in *Medicago truncatula*.

## Introduction

Anthocyanins and proanthocyanins (PAs) are two classes of flavonoids possessing benefits for human health (Butelli *et al.*, 2008; Lila *et al.*, 2016). In addition, the accumulation of anthocyanins can protect plants against various biotic and abiotic stresses, and provide colourful hues for plants to attract pollinators and seed dispersers (Zhang *et al.*, 2014; Saigo *et al.*, 2020). In forage crops, moderate accumulation of PAs protects ruminant animals from pasture bloat and improves the absorption of essential amino acids in the ruminant’s rumen (Dixon *et al.*, 2005).

The biosynthesis of anthocyanins and PAs shares a common upstream biosynthetic pathway (Dixon *et al.*, 2013). The proteins regulating their biosynthesis, namely the ternary MBW (MYB-bHLH-WD40) protein complexes, are conserved in higher plants. In this complex, the MYB transcription factor plays a crucial role in determining the activation of specific downstream genes to control the spatiotemporal accumulation of anthocyanins and PAs (Albert *et al.*, 2011; Dixon *et al.*, 2013; Yuan *et al.*, 2014; N. Lu *et al.*, 2021). Generally, activation or overexpression of these MYB activators alone, or coupled with a bHLH member, can strikingly promote anthocyanin and PA accumulation (Borevitz *et al.*, 2000; Butelli *et al.*, 2008; Peel *et al.*, 2009; Hancock *et al.*, 2012; Dixon *et al.*, 2013; Sun *et al.*, 2020; N. Lu *et al.*, 2021; Wang *et al.*, 2021). In the model legume *M. truncatula*, three anthocyanin biosynthetic MYB activators, LAP1, WHITE PETAL1 (WP1) and RED HEART1 (RH1), have been identified to regulate anthocyanin biosynthesis by activating anthocyanin biosynthetic genes (Peel *et al.*, 2009; Meng *et al.*, 2019; Wang *et al.*, 2021). Among them, WP1 and RH1 specifically regulate anthocyanin accumulation in petal and leaf, respectively (Meng *et al.*, 2019; Wang *et al.*, 2021). Constitutively or transiently overexpressing *LAP1* could massively induce the ectopic accumulation of anthocyanins in *M. truncatula* (Peel *et al.*, 2009; Picard *et al.*, 2013; Bond *et al.*, 2016).

Anthocyanins and PAs are believed to be synthesized on the cytoplasmic face of the endoplasmic reticulum (ER) by a conserved multi-enzyme complex that is anchored by the cytochrome P450 enzyme family members cinnamate 4-hydroxylase (C4H) and flavonoid 3´ hydroxylase (F3´H; Winkel, 2004; Zhao and Dixon, 2010), and are finally sequestered into the vacuole to prevent their oxidation in the cytoplasm, and to fulfil their function as pigments (Grotewold and Davies, 2008; Zhao and Dixon, 2010; Zhao, 2015). Compared with the well-studied transcriptional regulation mechanisms, the *in vivo* sequestration of anthocyanins and PAs is still poorly understood (Zhao, 2015), especially in legume species. Previously, two MULTIDRUG AND TOXIC COMPOUND EXTRUSION (MATE) transporters, *Medicago* MATE2 and MATE1, had been identified that participate in anthocyanin and PA sequestration (Zhao and Dixon, 2009; Zhao *et al.*, 2011). However, anthocyanins and PAs could still be detected in *mate2* and *mate1* mutants, indicating that there must be other members collaboratively involved in anthocyanin and PA sequestration (Zhao and Dixon, 2009; Zhao *et al.*, 2011). It has been accepted that two sequential steps, the delivery from the surface of the ER to the surrounding tonoplast, and the transmembrane transport across the tonoplast, are required for plants to eventually sequester anthocyanins into the vacuole (Grotewold and Davies, 2008; Zhao, 2015). To date, two kinds of non-overlapping transport models, vesicle-mediated trafficking and glutathione-S-transferase (GST)-mediated transport, have been proposed to be responsible for the first step of anthocyanin and PA transport (Grotewold and Davies, 2008; Zhao, 2015). Vesicle-mediated trafficking is supported by observations of the presence of anthocyanoplasts in the cytoplasm (Zhang *et al.*, 2006; Poustka *et al.*, 2007; Gomez *et al.*, 2011). The GST-mediated transport model is based on the characterization of BZ2 in maize and the identification of AtTT19 in *Arabidopsis thaliana* (Marrs *et al.*, 1995; Kitamura *et al.*, 2004). AtTT19 is a plant-specific *phi* class GST that is necessary for both anthocyanin and PA accumulation (Kitamura *et al.*, 2004). In *tt19* mutants, anthocyanin accumulation was not visible, and the seed coat colour was pale at the ripening stage due to the defect of anthocyanin and PA accumulation (Kitamura *et al.*, 2004; Sun *et al.*, 2012). In recent years, several TT19 homologs have been identified and characterized in many plants, all of which are considered to be essential for anthocyanin accumulation (Conn *et al.*, 2008; Hu *et al.*, 2016; Luo *et al.*, 2018; Jiang *et al.*, 2019; Wei *et al.*, 2019; Zhao *et al.*, 2019; Z. Lu *et al.*, 2021). Whether TT19-like GST is recruited to participate in PA accumulation in other plants is still unclear.

In this study, through analysis of previous transcriptomic data, expression pattern detection, phenotypic analysis of knockout mutants, and genetic complementation assays, we demonstrate that MtGSTF7, a TT19-like GST, is necessary for anthocyanin accumulation in the model legume *M. truncatula*. Finally, we show that LAP1 can bind to the *MtGSTF7* promoter to activate its expression, and that MtGSTF7 does not control PA accumulation, as does its Arabidopsis orthologue.

## Materials and methods

### Plant materials and growth conditions

*M. truncatula* wild type (WT) R108 seeds were obtained from the Noble Research Institute, Ardmore, OK, USA. The *mtgstf7* mutants were ordered from the *M. truncatula Tnt1* mutant database (https://medicago-mutant.dasnr.okstate.edu/mutant/index.php) with the numbers NF2357 and NF10672. For greenhouse cultivation, the seeds were gently scarified by rubbing them between two pieces of sandpaper, then transferred to water in petri dishes and placed at 4 °C, in the dark for 3–5 d to allow germination. To obtain sterile plants, the *M. truncatula* seeds were soaked in concentrated anhydrous sulfuric acid in a 2 ml Eppendorf tube and then shaken for 6–8 min to scarify the seed coat. After removing the H_2_SO_4_ and rinsing the seeds with chilled water 3–5 times, 3% (v/v) sodium hypochlorite solution was added to sterilize the scarified seeds for 8–10 min.

Plants used in this study were grown under controlled greenhouse conditions with a mean temperature of 22 °C, 16 h/8 h photoperiod and suitable humidity. For induced anthocyanin accumulation, 3-day-old seedlings were grown under high intensity light (HL) at an average of 252-270 µmol m^-2^ s^-1^ for 4 d. Light under control (CT) conditions was 126-135 µmol m^-2^ s^-1^. The light intensity was measured with a Digital Lux Meter (SMART SENSOR, AS813, China).

### Comparative analysis of the transcriptomic datasets

The normalized transcriptomic data of Peel *et al.* (2009) were downloaded from ArrayExpress (http://www.ebi.ac.uk/microarray-as/ae/) with the accession number E-MEXP-1854. Firstly, the microarray IDs were converted to V4.0 gene IDs based on the mapping file (Supplementary Table S1). Then, the significant DEGs (differentially expressed genes) induced by LAP1 were screened out based on the log_2_FC values (FC value = RMA_LAP1_/RMA_GUS_; RAM is the normalized value analysed by Peel *et al.*, 2009) ≧1 and *P* value≧0.05; (Supplementary Table S2). The transcriptomic dataset of Bond *et al.* (2016) was downloaded from (https://static-content.springer.com/esm/art%3A10.1186%2Fs13007-016-0141-7/MediaObjects/13007_2016_141_MOESM1_ESM.xlsx) and the original data were downloaded from the Sequence Read Archive (SRA) database with accession number SRP091342. In the released list of significant DEGs by Bond *et al.*, 2016, values for each individual data set were log_2_-transformed normalized counts (Supplementary Table S3). To avoid the deviation caused by different sequencing and analytical techniques, we used the log_2_FC values of DEGs for further analysis. The DEG IDs and their corresponding log_2_FC values in Peel *et al.* (2009) and Bond *et al.* (2016) datasets (Supplementary Table S4) were used to plot the Venn diagram and clustering heatmap using the TBtools software (Chen *et al.*, 2020).

### Gene expression atlas analysis

Expression atlases of target genes were retrieved from the previously released processed GeneChip Array data (https://www.ebi.ac.uk/arrayexpress/files/E-MEXP-1097/E-MEXP-1097.processed.1.zip;Benedito *et al.*, 2008). The corresponding probe IDs of target genes were queried in the mapping file (Supplementary Table S5) and the tissue expression of genes was displayed with the average values of Affymetrix: CHPSignal.

### Observation of anthocyanin autofluorescence

Images were captured with a laser scanning confocal microscope (TCS SP8 X, Leica, Germany). Anthocyanin autofluorescence was captured at the emission spectrum between 610–650 nm after excitation at 541 nm.

### Pigment extraction, HPLC and measurement of anthocyanins

Accurately weighed tissues were ground into powder in 1.5 ml tubes after freezing in liquid nitrogen, and pigment extraction solution (methanol containing 0.1% HCl) was added. After vortexing and centrifugation, the residues were re-extracted until the supernatants were colourless. The supernatants were pooled as crude extract for the following analysis.

For HPLC analysis, an equal volume of double-deionized water and chloroform was added to the pooled supernatant to remove the chlorophyll. After centrifugation, the aqueous supernatant was dried in a vacuum freeze drier, and the dried samples were resuspended in methanol for HPLC separation. HPLC separation was performed on an Agilent LC1260 infinity II HPLC system (Agilent Technologies, USA) with a reverse phase, Biphenyl, 5 μm, 250 × 4.6 mm column (Phenomenex Kinetex, USA) and elution with solution A (0.2% phosphoric acid) and solution B (acetonitrile) at 1 ml min^-1^ flow rate. The elution gradient was as follows: 0–2 min, 5–10% solution B; 2–10 min, 10–18% solution B; 10–14 min, 18–20% solution B; 14–18 min, 20–22% solution B; 18–22 min, 22–40% solution B; 22–24 min, 40–100% solution B. The absorbance was detected at 530 nm. To measure the anthocyanin content, cyanidin chloride was used to construct a standard curve and the anthocyanin content was calculated as standard equivalents.

### Analysis of PAs

To qualitatively estimate PA content, mature seeds were stained overnight in 0.1% (w/v) DMACA reagent (p-dimethylaminocinnamaldehyde dissolved in methanol-3N HCl) and then washed with ethanol: acetic acid (75:25) three times before observation.

The extraction and quantitative detection of PAs were performed as described previously (Liu *et al.*, 2016; Jun *et al.*, 2021). Briefly, about 0.05 to 0.1 g dry seeds were accurately weighed and ground into powder in liquid nitrogen. The powders were extracted four times with 6 ml PA extraction solution (70% acetone/0.5% acetic acid, v/v) by vortexing and sonicating at room temperature (~ 25–30 °C) for 30 min until the residues were white. The residues (containing insoluble PAs) were lyophilized and the pooled supernatants were further extracted three times with equal volumes of chloroform, and twice with hexane. The resulting aqueous phase (containing soluble PAs) was lyophilized and resuspended in 100 µl PA extraction solution.

The soluble PA content was measured by the DMACA method. For this, 1 µl soluble PA was made to react with 0.2% (w/v) DMACA regent, and the absorbance at 640 nm was recorded after 5 min. Epicatechin was used as the standard to construct a standard curve, and the absorbance values were converted into epicatechin equivalents. The insoluble PA content was quantified by the butanol/HCl method as described previously (Pang *et al.*, 2007). Standard curves of epicatechin and procyanidin B1 are shown in Supplementary Fig. S1.

### Total RNA extraction, RT–PCR, and qRT–PCR

Total RNA from the infiltrated leaves was extracted to detect the expression of genes in the transient expression assay. The total RNA from different tissues of WT was extracted to detect the tissue expression pattern of *MtGSTF7* in *M. truncatula*. The total RNA from hypocotyls of WT and *mtgstf7* mutants was extracted to detect the expression of *MtGSTF7* and anthocyanin biosynthesis genes. The total RNA from transgenic *G. max* hairy roots and leaves was extracted to detect the expression of soybean genes. The total RNA was isolated using an RNA simple Total RNA Kit (Tiangen, China) according to the manufacturer’s protocol, and 2 µg total RNA was subsequently used for reverse transcription. The first-strand cDNAs were reverse transcribed using the HiScript® II 1st Strand cDNA Synthesis Kit (+gDNA wiper; R212; Vazyme, China). The cDNAs corresponding to 200 ng RNA were then used as templates for qRT–PCR and RT–PCR. The relative transcript levels of genes were calculated by the 2^-∆Ct^ method. *MtACTIN* and *MtGAPDH* were used as internal reference genes for analysing the qRT–PCR data for *M. truncatula*. *AtEF1a* and *AtACTIN2* was used as internal reference gene for analysing the qRT–PCR data for *A. thaliana*. *GmCONS4* (*CONSTITUTIVE GENES 4*) and *GmACTIN* was used as internal reference gene for analysis of the qRT–PCR data in *G. max.* For RT–PCR, 32 cycles were carried out to detect the gene transcripts. Primer information is listed in Supplementary Table S6.

### Plasmid construction and plant transformation

Complete coding sequences and different length *MtGSTF7* promoters were amplified from WT cDNA and genomic DNA using Phanta Max Super-Fidelity DNA Polymerase (P505, Vazyme, China) and purified using the EasyPure Quick Gel Extraction Kit (EG101, TransGen, China). All of the plasmids were constructed using the ClonExpress II One Step Cloning Kit (C112, Vazyme, China) and all the final constructs were confirmed by Sanger sequencing.

For analysis of the sub-cellular localization of MtGSTF7, the *MtGSTF7* coding sequence without the stop codon was inserted into the pYS22 vector between the *Xho*I and *Kpn*I restriction enzymes sites to generate the pro35S::cMtGSTF7-GFP construct. The construct was transformed into *Agrobacterium tumefaciens* EHA105 strain to be introduced into *Nicotiana benthamiana* leaves via the underside of the leaf. The pYS22 vector was used as the positive control. GFP and MtGSTF7-GFP fluorescence was examined using a confocal laser scanning microscope (FV1000, Olympus, Japan).

For complementation of Arabidopsis *tt19* mutants, the pCAMBIA3301 vector was digested by *Nco*I and *Bst*EII (*Eco*91I), and the complete *MtGSTF7* coding sequence was cloned into the linearized vector to generate the pro35S::cMtGSTF7 construct. The construct was then transformed into *A. tumefaciens* EHA105 strain and introduced into *tt19-7* and *tt19-8* via the floral dip method. The positive transgenic plants were screened by spraying with 20 mg l^-1^ Basta and confirmed by PCR analysis.

For genetic complementation of *MtGSTF7*, a 8070 bp MtGSTF7 genomic sequence (with a 4417 bp native promoter sequence and a 1500 bp 3´ untranslated region) was amplified using the Tks Gflex™ DNA Polymerase (R060, Takara, Japan). The genomic sequence of *MtGSTF7* was inserted into the *Hin*dIII and *Eco*91I linearized pCAMBIA3301 vector to construct the proMtGSTF7::gMtGSTF7-3’UTR construct. The construct was then transformed into the *A. tumefaciens* EHA105 strain for stable transformation into *mtgstf7-1*, as described previously (Cosson *et al.*, 2006). The positive transgenic plants were confirmed by genotyping and RT–PCR for the presence of *MtGSTF7*. All primers sequences are listed in Supplementary Table S6.

### Transient gene expression in *M. truncatula*

The complete *LAP1* coding sequence was cloned into the pCAMBIA3301 vector to create the pro35S::cLAP1 construct, which was transferred into *A. tumefaciens* strain EHA105. The primer sequence is listed in Supplementary Table S6. The transformed *A. tumefaciens* was inoculated into LB liquid medium with 100 µg ml^-1^ kanamycin and incubated at 28 °C on a shaker overnight to reach OD_600_ of 1.0 to 2.0. The culture was centrifuged at 4500 ×*g* for 10 min and the pellet re-suspended in infiltration buffer (10 mM NaCl, 1.75 mM CaCl_2_, 2 µl Tween-20, 100 mM acetosyringone) to OD_600_ of 1.0 to 1.2. The third to fifth fully unfolded trifoliate leaves of 3–4 week-old healthy *M. truncatula* plants were selected for infiltration.

### Yeast one-hybrid (Y1H) assay

To generate the prey vector AD-LAP1, the complete coding sequence of *LAP1* was inserted into the pGADT7.1 vector (Clontech, Japan) between the *Bam*HI and *Sma*I restriction enzymes sites. To construct the bait vector pABAi-proMtGSTF7, a 1982 bp *MtGSTF7* upstream promoter sequence was spliced by overlap extension PCR to remove an 18 bp sequence that presents the *Bbs*I restriction enzyme sites. Subsequently, the spliced sequence was cloned into the pABAi vector (Clontech). The Y1H assay was carried out according to the manufacturer’s instructions of the Matchmaker® Gold Yeast One-Hybrid System (PT4087, Clontech) and the Yeastmaker™ Yeast Transformation System 2 (PT1172, Clontech). Briefly, the bait vector was digested with *Bbs*I and transformed into yeast strain Y1HGold to create the bait reporter strain. After confirmation by PCR, the positive bait reporter strain was grown on SD/-Ura media with different concentrations of Aureobasidin A (AbA) (50, 100, 200 and 300 ng ml^-1^) to determine the suitable inhibitory concentration. Then, the prey vector was transformed into the positive bait reporter strain to test the bait/prey interactions. The empty pABAi and pGADT7.1 vectors were used as controls. The primers used are listed in Supplementary Table S6.

### Dual-luciferase reporter assay

A 1924 bp *MtGSTF7* upstream promoter sequence was cloned into the pGreenII 0800-LUC vector between the *Pst*I and *Nco*I restriction enzyme sites to create the reporter vector proMtGSTF7::LUC. After sequence confirmation, proMtGSTF7::LUC was transformed into *A. tumefaciens* strain GV3101 that contained the pSoup helper plasmid. The pro35S::cLAP1 and the empty vector pro35S::GUS (pCAMBIA3301) were used as the effector vectors. The *A. tumefaciens* reporter and effector strains were mixed at a ratio to 1:9 to infiltrate into *N. benthamiana* leaves, as described previously (Hellens *et al.*, 2005). The luciferase activity was detected 2 d after infiltration. The primers sequences are listed in Supplementary Table S6.

To analyse the luciferase activity, 1 mM D-luciferin, potassium salt (D8390, Solarbio, China) was evenly sprayed on the surface of *N. benthamiana* leaves. After reaction in the dark for 5–10 min, the fluorescence was captured. For quantitative detection analysis, the activities of firefly and Renilla luciferase were quantified using the Dual-Luciferase® Reporter Assay System (E1910, Promega, USA) following the manufacturer’s instructions, and luminescence values were measured using a multifunctional microplate reader (SpectraMax iD3, Molecular Devices, USA).

## Results

### *MtGSTF7* is activated by LAP1 and its expression correlates with anthocyanin accumulation in *M. truncatula*

**Fig. 1.**
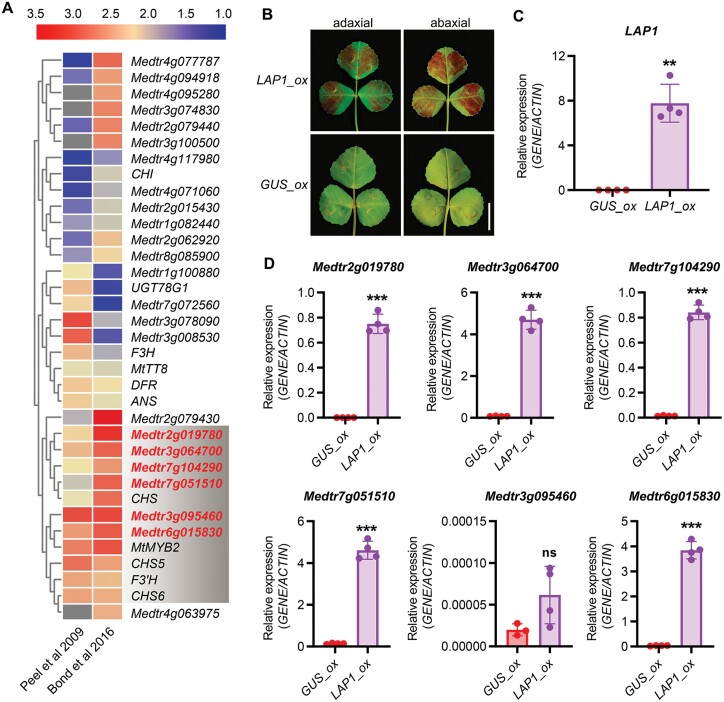
Five common genes are highly induced in *LAP1* overexpressing leaves. (A) The clustered heatmap based on the fold change values of 35 common DEGs. Genes in the grey-shaded region were highly induced (log_2_FC≧3) by overexpressing *LAP1*. (B) The adaxial and abaxial sides of ecotype R108 WT leaves transiently overexpressing *LAP1* (*LAP1_ox*) and GUS (*GUS_ox*). The *GUS* gene was used as a negative control (*GUS_ox*). The experiment was conducted with three independent repeats with similar results. (C) The relative transcript levels of *LAP1* in *LAP1_ox* and *GUS_ox* leaves determined by qRT–PCR (**P<0.01, two-tailed Welch’s *t*-test). The data are mean values ±SD (*n*=4). Similar results were acquired with two independent biological replicates. (D) The relative transcript levels of genes marked by red letters in the grey-shaded region in (A) verified by qRT–PCR (***P<0.001; ns, *P*>0.05; two-tailed Welch’s *t*-test).

Two independent experiments showed extremely high level accumulation of anthocyanins in leaves overexpressing *LAP1* (Peel *et al.*, 2009; Bond *et al.*, 2016), associated with the expression of anthocyanin-related genes. To further screen and identify anthocyanin accumulation-related genes, we compared two previously released transcriptome datasets (Peel *et al.*, 2009; Bond *et al.*, 2016) derived from leaves overexpressing *LAP1* and focused on the common DEGs (|log_2_FC|≧1 and *P* value≦0.05). According to this comparison, we found 35 DEGs that were shared between these two transcriptomic datasets (Supplementary Fig. S2). A heatmap clustered by log_2_FC values of 35 common DEGs showed that 14 DEGs were remarkably up-regulated (log_2_FC≧3) in those two transcriptomic datasets, indicating that all of these genes were greatly induced by overexpressing *LAP1* (Fig. 1A; Table 1). Apart from the six biosynthetic genes (*CHS5*, *CHALCONE SYNTHASE 5*; *CHS6*, *CHALCONE SYNTHASE 6*; *CHS*, *CHALCONE SYNTHASE*; *F3´H*, *FLAVONOID 3’-HYDROXYLASE*; *DFR*, *DIHYDROFLAVONOL REDUCTASE*; and *ANS*, *ANTHOCYANIDIN SYNTHASE*), and two transcription factors (TFs) identified previously (MtMYB2 and MtTT8), a further six genes (*Medtr2g019780*, *Medtr3g064700*, *Medtr7g104290*, *Medtr7g051510*, *Medtr3g095460*, and *Medtr6g015830*) caught our attention, and were selected for subsequent analysis (Fig. 1A; Table 1).

**Table 1.** The information of genes extremely activated by LAP1 (log_2_FC≧3 in two transcriptomic datasets).

Gene IDs	log_2_FC in Peel *et al* (2009)	log_2_FC in Bond *et al* (2016)	Annotation
Medtr3g095460	7.7006817	7.838319394	nodulin MtN21/EamA-like transporter family protein
Medtr3g083910 (CHS5, chalcone synthase 5)	6.589193019	5.260457267	chalcone and stilbene synthase family protei
Medtr5g079670 (MtMYB2)	6.077671733	7.443995756	myb transcription factor
Medtr6g015830	5.4169854	7.264546709	malonyl-CoA:isoflavone 7-O-glucoside malonyltransferase
Medtr3g083920 (CHS6, chalcone synthase 6)	5.213524554	4.920912433	chalcone and stilbene synthase family protein
Medtr4g109470 (F3’H, flavonoid 3’-hydroxylase)	5.1542956	4.614758629	flavonoid hydroxylase
Medtr3g064700	4.695889167	7.043531511	glutathione S-transferase, amino-terminal domain protein
Medtr1g022445 (DFR, dihydroflavonol reductase)	4.3313268	3.881723211	dihydroflavonol 4-reductase
Medtr5g011250 (ANS, anthocyanidin synthase)	4.264772867	3.394797924	leucoanthocyanidin dioxygenase-like protein
Medtr2g019780	4.122005946	8.7663448	auxin-binding protein ABP19b
Medtr7g104290	3.822662247	5.490588201	UAA transporter family protein
Medtr5g007730 (CHS, chalcone synthase)	3.492184967	6.646533624	chalcone and stilbene synthase family protein
Medtr1g072320 (MtTT8)	3.4294313	3.218784057	bHLH transcription factor
Medtr7g051510	3.0701301	7.023407627	glycoside hydrolase family 1 protein

To verify previous transcriptomic data, we transiently overexpressed *LAP1* in WT leaves. The results showed that sites around *Agrobacterium* infection areas turned red compared with the corresponding controls (Fig. 1B, C). Subsequent qRT-PCR analysis revealed that the transcript levels of *Medtr2g019780*, *Medtr3g064700*, *Medtr7g104290*, *Medtr7g051510*, and *Medtr6g015830* were significantly higher (*P*<0.001) in leaves overexpressing *LAP1* than those in leaves overexpressing *GUS* (Fig. 1D), suggesting that these five genes are most likely to participate in anthocyanin accumulation.

**Fig. 2.**
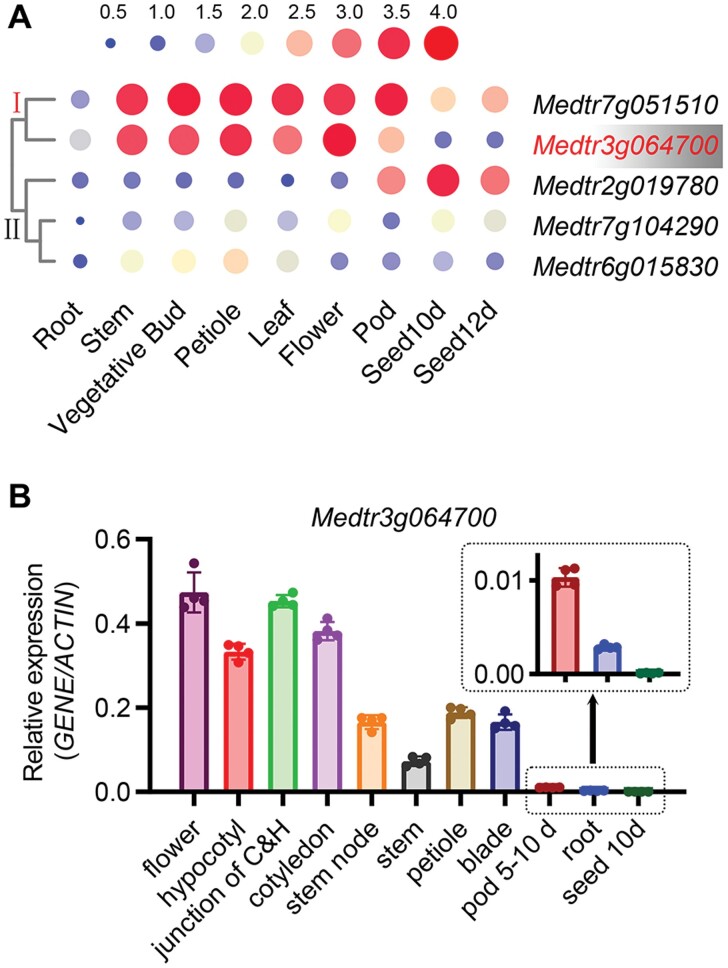
The *Medtr3g064700* gene is expressed in anthocyanin accumulating organs. (A) The gene expression atlas of *Medtr3g064700*, *Medtr7g051510*, *Medtr7g104290*, *Medtr2g019780*, and *Medtr6g015830* in different organs of *M. truncatula*. The normalized expression values were log_10_ base transformed. The scale is shown at the top. Different sizes and colours in the scale indicate the expression amount. ‘Ⅰ’ and ‘Ⅱ’ show the two different sub-groups clustered by the tissue expression pattern of genes. Genes in cluster ‘Ⅰ’ showed a higher expression in anthocyanin accumulation tissues. (B) The tissue expression pattern of *Medtr3g064700* determined by qRT–PCR. Junction of C&H, the junction region of cotyledons and hypocotyl. Two independent biological replicates showed similar results.

Retrieving the *M. truncatula* microarray data from the gene expression atlas (https://www.ebi.ac.uk/arrayexpress/experiments/E-MEXP-1097/ and https://medicago.toulouse.inrae.fr/MtExpress), we found that the expression patterns of the five candidate genes were separated into two obvious ­clusters (Fig. 2A). Genes in cluster ‘Ⅰ’ showed a high expression in stem, vegetative buds, petiole, leaf, flower and pod, where anthocyanins are usually accumulated (Fig. 2A). Moreover, the *Medtr7g051510* in cluster ‘Ⅰ’ also showed a relatively higher expression in seeds where anthocyanins are not accumulated (Fig. 2A). So, we focused on *Medtr3g06470* and further verified its tissue expression pattern by qRT–PCR. The result was similar to the microarray data that *Medtr3g06470* was expressed at flower, hypocotyl, junction of cotyledon, cotyledon, stem node, stem, petiole blade, and pod where anthocyanins accumulated (Fig. 2B). In root and seeds, the transcript of *MtGSTF7* could almost not be detected (Fig. 2B).

### MtGSTF7 (Medtr3g064700) is necessary for anthocyanin deposition in *M. truncatula*

**Fig. 3.**
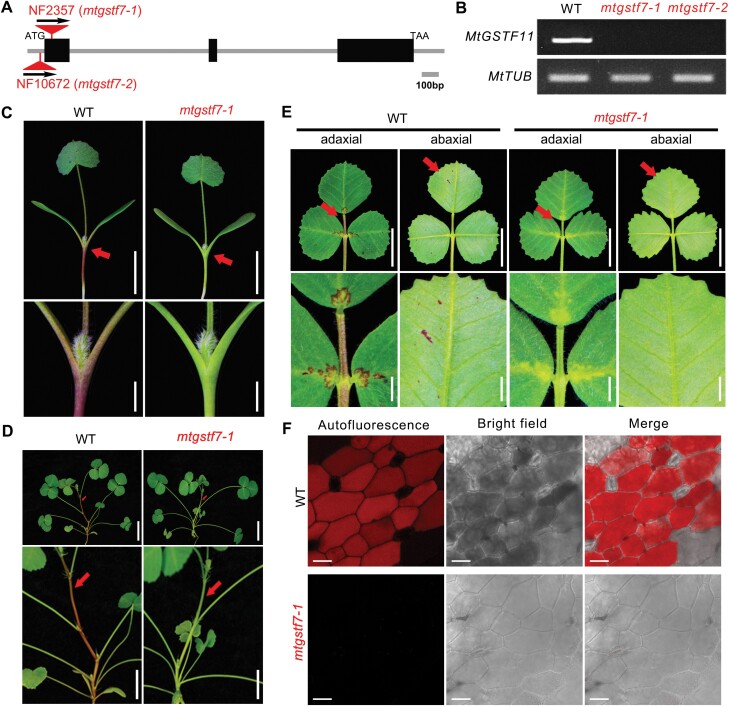
Defective anthocyanin accumulation in the *mtgstf7-1* mutant. (A) Diagram showing the gene structure of *MtGSTF7* and the *Tnt1* insertions in mutant alleles. Blank boxes indicate exons and lines between them represent introns. Vertices of red triangles denote *Tnt1* insertion sites. Arrows indicate the orientations of *Tnt1* insertions. (B) Transcript abundance of *MtGSTF7* in WT and two *mtgstf7* mutants determined by RT–PCR. The transcript abundance of *MtTUB* was used as the internal control. (C) Phenotypes of the WT and *mtgstf7-1* seedlings. Red arrows indicate the junctions between cotyledon and hypocotyl. Magnified images of junctions are shown in the lower panel. Scale bars: upper, 1 cm; lower, 2 mm. (D) Phenotype of 45-day-old plants of WT and *mtgstf7-1* mutant. Arrows indicate the red and green stems in WT and *mtgstf7-1* mutant, respectively. The magnified images are shown in the lower panel. Scale bars: upper, 1 cm; lower, 5 mm. (E) Phenotypes of WT and *mtgstf7-1* leaves. Images in the upper panel show the adaxial and the abaxial sides of leaves. Red arrows indicate the spots accumulating anthocyanins in the leaflets, and the magnified images are shown in the lower panel. Scale bars: upper, 1 cm; lower, 2 mm. (F) Anthocyanin autofluorescence captured from the adaxial side of WT and *mtgstf7-1* mutant leaflets. Scale bars: 25 µm.

*Medtr3g064700* encodes a typical GST protein with a canonical βαβαββα-N-terminal domain and a C-terminal domain composed of an α helix (Supplementary Fig. S3). According to the previously identified GST family members, *Medtr3g064700* is named as *MtGSTF7* (Hasan *et al.*, 2021). To further functionally characterize MtGSTF7, we used the *MtGSTF7* genomic sequence with a partial promoter and with the *Tnt1* FSTs (flanking sequence tags) as a query for BLAST analysis in the *M. truncatula* mutant database (https://medicago-mutant.dasnr.okstate.edu; Tadege *et al.*, 2008; Sun *et al.*, 2019), and successfully isolated two independent *Tnt1* insertion lines of *MtGSTF7*, NF2357 (*mtgstf7-1*) and NF10672 (*mtgstf7-2*). According to PCR and Sanger sequencing, we confirmed that*Tnt1* retrotransposons were separately inserted in the first exon (45 bp downstream of ATG) and the 5´-UTR (35 bp upstream of ATG) of *MtGSTF7* in *mtgstf7-1* and *mtgstf7-2*, respectively (Fig. 3A). Through RT–PCR, we could not detect the full-length transcript of *MtGSTF7* in *mtgstf7-1* and *mtgstf7-2*, indicating that *mtgstf7-1* and *mtgstf7-2* are null mutants (Fig. 3B). Because a similar phenotype was observed between *mtgstf7-1* and *mtgstf7-2* during the entire developmental period, the *mtgstf7-1* mutant allele was used for further analysis in the following studies.

In WT seedlings, the junction of cotyledons and hypocotyl, and the bases of cotyledons and petioles are red due to the deposition of anthocyanins (Fig. 3C). However, the anthocyanin depositions were not visible in *mtgstf7-1* mutant seedlings (Fig. 3C). The anthocyanin content was decreased sharply so as to be undetectable in extracts from fresh hypocotyls of the *mtgstf7-1* mutant (Supplementary Fig. S4). In addition, the disappearance of anthocyanins was also observed in other organs, including leaves, petioles, stems, and even seedpod spines (Fig. 3D, E; Supplementary Fig. S5). Analysis of anthocyanin autofluorescence showed that the deposition of anthocyanins was almost absent in *mtgstf7-1* vacuoles (Fig. 3F).

**Fig. 4.**
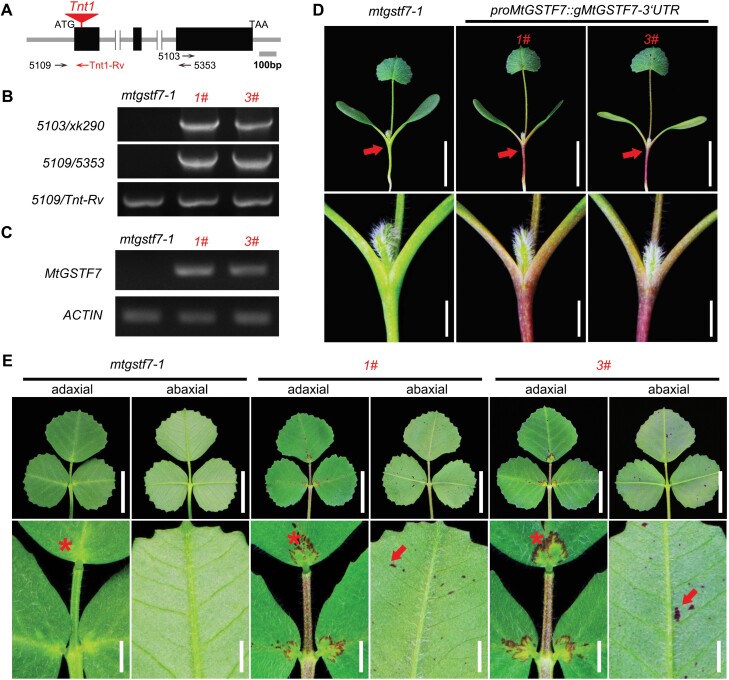
The anthocyanin deficiency of *mtgstf7-1* can be completely rescued by *MtGSTF7*. (A) Diagram showing the *MtGSTF7* gene structure and the locations of primers used for genotyping. Arrows indicate the orientations of primers. (B) The genotyping of independent rescued lines. Primer xk290 is a specific reverse primer located in the *ProMtGSTF7::gMtGSTF7-3’UTR* construct. The locations of other primers are shown in (A). (C) Transcript abundance of *MtGSTF7* in *mtgstf7-1* and two rescued lines determined by RT–PCR. *MtACTIN* was used as an internal control. (D) Phenotypes of *mtgstf7-1* and two representative independent rescued lines. Red arrows indicate the junctions between cotyledon and hypocotyl, and magnified images are shown in the lower panel. Scale bars: upper, 1 cm; lower, 2 mm. (E) Leaf phenotypes of *mtgstf7-1* and two independent rescued lines. Images in the upper panel show the adaxial and the abaxial sides of leaves. Images in the lower panel show the magnification of the adaxial side of the leaflet basal region and the abaxial side of the terminal leaflets. Asterisks indicate that the disappearance of anthocyanin deposition on the adaxial side of the leaflets was complemented by introducing the *MtGSTF7* genomic sequence into the *mtgstf7-1* mutant. Red arrows indicate the scattered spots on the abaxial side of terminal leaflets of rescued lines. Scale bars: upper, 1 cm; below, 2 mm.

To verify the *mtgstf7-1* mutant phenotype, we performed backcrossing and genetic linkage analysis. The segregation ratio of WT: mutants (139:42) in the F_2_ population was in agreement with Mendel’s law of segregation (Supplementary Table S7). Besides, all 42 isolated mutants showed similar phenotypes with anthocyanin deficiency, and these mutants co-segregated with the homozygous *Tnt1* insertion in *MtGSTF7* (Supplementary Fig. S6). To test whether *MtGSTF7* could complement the mutant phenotype, we performed a genetic complementation experiment by transferring the *MtGSTF7* genomic fragment into the mutant background and obtained ten independent transgenic plants. Genotyping and genetic analysis showed that seven of the transgenic lines carried the *MtGSTF7* genomic fragment in the *mtgstf7-1* genetic background (Fig. 4A, B), and RT–PCR could amplify the entire transcript of *MtGSTF7* in these rescued lines (Fig. 4C). The defect of anthocyanin accumulation seen in the mutant was completely recovered in these positive transgenic lines (Fig. 4D, E; Supplementary Fig. S4). On the basis of these results, we conclude that *MtGSTF7* is essential for anthocyanin accumulation in *M. truncatula*.

To test whether mutation of *MtGSTF7* affects the expression of anthocyanin biosynthetic genes, we checked the expression of anthocyanin-related genes by qRT–PCR. The results showed that the transcript levels of all detected genes in the *mtgstf7-1* mutant were not significantly different (*P*>0.05) from that in WT, except for a slight increase in *F3´H* transcript (Supplementary Fig. S7), indicating that the anthocyanin biosynthetic process seemed to not be affected by mutation of *MtGSTF7*.

### Ectopic accumulation of anthocyanins is blocked by loss of function of *MtGSTF7*

**Fig. 5.**
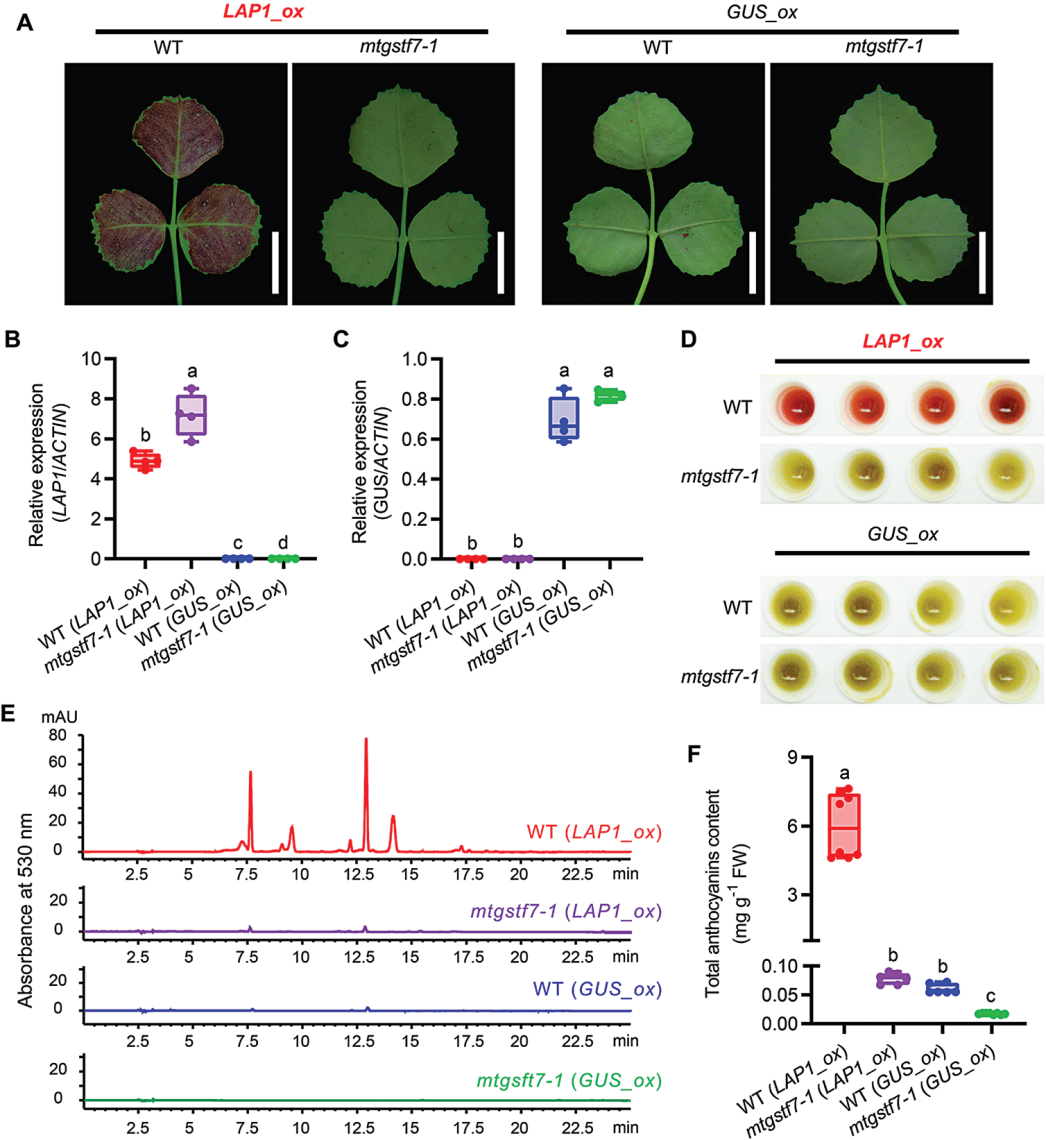
LAP1-induced anthocyanin accumulation depends on MtGSTF7 in *M. truncatula.* (A) The abaxial sides of WT and *mtgstf7-1* leaves that transiently overexpress *LAP1* (*LAP1*_ox). The transient overexpression of *GUS* (*GUS*_ox) was used as the negative control. Scale bars: 1 cm. (B) Relative transcript levels of *LAP1* in leaves that transiently overexpress *LAP1* or *GUS*. (C) Relative transcript levels of *GUS* in leaves that transiently overexpress *LAP1* or *GUS*. (D) The pigments extracted from leaves which transiently overexpress *LAP1* or *GUS*. Red colours in the extracts are the results of anthocyanin accumulation and the yellowish-green colours are the chlorophyll. Four independent leaves from different plants were extracted for each experimental group. (E) Reverse-phase HPLC chromatograms of anthocyanins extracted from WT and *mtgstf7-1* leaves that transiently overexpress *LAP1* and *GUS*. (F) The total anthocyanin contents of WT and *mtgstf7-1* leaves that transiently overexpress *LAP1* and *GUS*. The anthocyanin content was calculated as cyanidin chloride equivalents. FW, fresh weight. The data are mean values ±SD (*n*=4). Gene transcript levels in (B) and (C) were determined by qRT–PCR. *MtACTIN* was used as the internal control. The data are mean values ±SD (*n*=4). Different letters in (B), (C) and (F) donate significant differences (*P*<0.05; two-way ANOVA tests).

Previous studies demonstrated that transient overexpression of *LAP1* in *M. truncatula* leaves could dramatically induce anthocyanin accumulation (Peel *et al.*, 2009; Picard *et al.*, 2013; Bond *et al.*, 2016). To investigate whether the loss of function of *MtGSTF7* affects ectopic accumulation of anthocyanins, we transiently overexpressed *LAP1* in WT and *mtgstf7-1* leaves. Similar to the previous reports utilizing stable transformation (Pang *et al.*, 2009), the transient overexpression of *LAP1* induced a massive accumulation of dark red anthocyanins in the infection areas of WT leaves (Fig. 5A, B; Supplementary Fig. S8A), whereas, in *mtgstf7-1* leaves, the infection areas remained green (Fig. 5A-C; Supplementary Fig. S8A). The same conclusion was reached by comparison of the crude extracted leaf pigments (Fig. 5D). Reverse-phase HPLC directly showed that the *LAP1*-induced anthocyanin content was remarkably reduced in *mtgstf7-1* compared with WT (Fig. 5E), by 50–100-fold (Fig. 5F). Quantification of anthocyanin biosynthetic gene transcripts revealed that the transcript abundance of several anthocyanin biosynthesis genes, especially most of the late biosynthesis genes, was higher in *mtgstf7-1* mutant overexpressing *LAP1* than in WT overexpressing *LAP1* (Supplementary Fig. S8B). Taken together, all of these results indicated that loss of function of *MtGSTF7* led to the drastic reduction of anthocyanin accumulation.

**Fig. 6.**
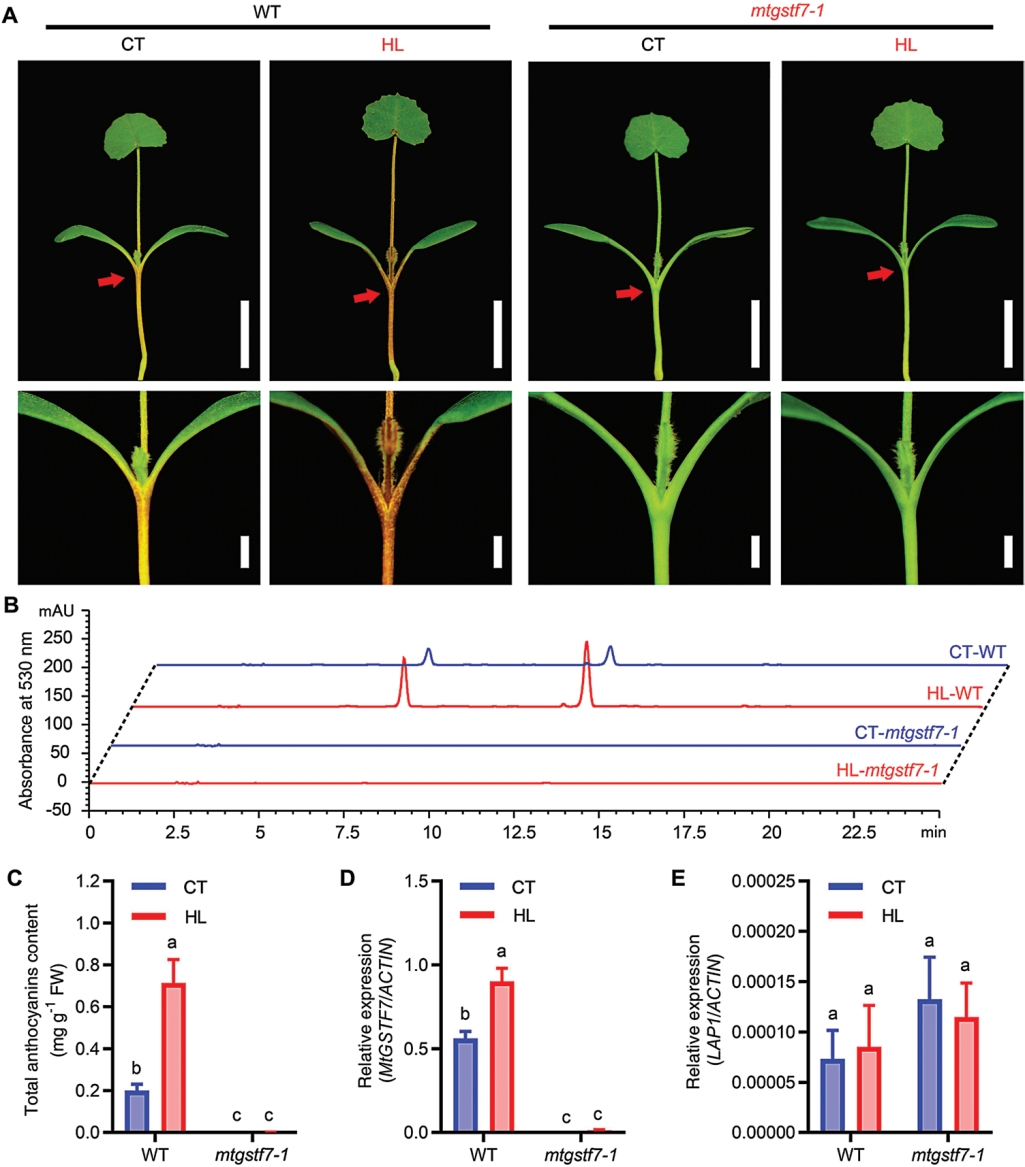
The light-induced accumulation of anthocyanins relies on MtGSTF7. (A) Phenotypes of WT and *mtgstf7-1* seedlings in high light conditions. CT, control intensity of lights; HL, high intensity of lights. Red arrows indicate the junctions between cotyledon and hypocotyl (upper panel), and the magnified images are shown in the lower panel. Scale bars: upper, 1 cm; lower, 2 mm. (B) Reverse-phase HPLC chromatograms of anthocyanins extracted from WT and *mtgstf7-1* seedlings exposed to different intensity lights. (C) The quantification of anthocyanin contents in WT and *mtgstf7-1* seedlings treated under control (CT) and high intensity (HL) light. (D) The relative transcript level of *MtGSTF7* in WT and *mtgstf7-1* under control (CT) and high intensity (HL) of light. (E) The relative transcript levels of *LAP1* in WT and *mtgstf7-1* under control (CT) and high intensity (HL) of light. Transcript levels were normalized against *MtACTIN*. Two independent experiments showed similar results. Different letters denote significant differences between each other (*P*<0.01, two-way ANOVA tests).

Anthocyanin accumulation can be induced by different abiotic or biotic stresses, such as low temperature, insufficient salt, and high intensity light (Das *et al.*, 2012; Sharma *et al.*, 2019). To investigate whether the accumulation of anthocyanins can be induced by stress in the *mtgstf7-1* mutant, 3-day-old seedlings of WT and *mtgstf7-1* were treated with HL for 4 d. Anthocyanin accumulation was notably increased after treatment by HL stress in WT seedlings (Fig. 6A). The HPLC chromatogram and the quantification of anthocyanin contents revealed that the accumulation of anthocyanins was increased to 3–4 fold in WT under HL stress (Fig. 6B, C). The qRT–PCR results showed that the expression of *MtGSTF7* was significantly induced (*P*<0.01) in WT by HL stress (Fig. 6D). However, in *mtgstf7-1*, the accumulation of anthocyanins and the expression of *MtGSTF7* was barely detected under HL conditions (Fig. 6A-D). The above results demonstrated that *MtGSTF7* is activated by HL stress and is essential for anthocyanin accumulation. In comparison with *MtGSTF7*, the transcript of *LAP1* was present at low abundance and was not induced by HL, neither in WT or in *mtgstf7-1* (Fig. 6E), indicating that the HL-induced anthocyanin accumulation occurred independent of *LAP1*.

### LAP1 can bind to the *MtGSTF7* promoter to activate its transcriptional activity

**Fig. 7.**
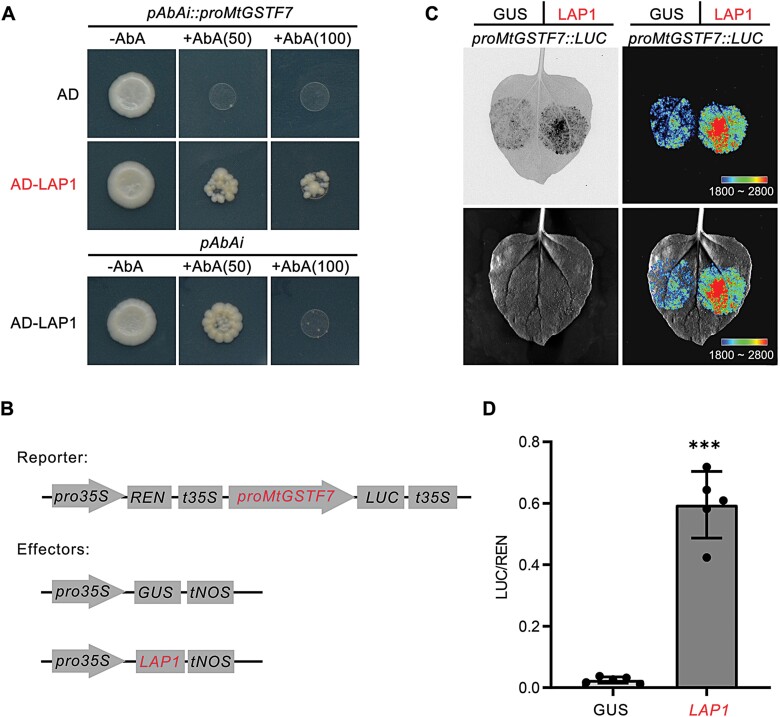
LAP1 can bind to the *MtGSTF7* promoter to activate its expression. (A) Yeast-one-hybrid assay showing the interaction of LAP1 and *MtGSTF7* promoter. AD represents the empty pGADT7.1 vector. Numbers in brackets indicate the concentration of Aureobasidin A (AbA), ng ml^-1^. (B) Schematic diagrams showing the reporter and effector constructs used for promoter-luciferase reporter assays. The effector of GUS was used as the negative control. (C) The promoter-luciferase assays showing activation of the *MtGSTF7* promoter by LAP1. The upper left image shows the original chemiluminescence picture after 150 s of exposure. The upper right image shows the chemiluminescence image embellished with pseudo-colours. The lower left image shows the monochrome picture of the infiltrated tobacco leaf. The lower right image shows the merged image of the infiltrated tobacco leaf and pseudo-colour picture. Similar results were obtained with five independent biological replicates. (D) The quantitative result of dual-luciferase assays. Ratio of LUC and REN represents the activation efficiency of effectors (GUS and LAP1) when co-transformed with the same reporter. (***P<0.001, unpaired two-tailed Welch’s *t*-test). The data are mean values ±SD from five independent biological replicates. Four technical replicates were performed for each biological replicate.

Analysis of the 2.0 kb upstream sequence from the initiation codon of *MtGSTF7* in the PlantCARE database (Lescot *et al.*, 2002) showed that six MYB binding sites were present, located at –1902, –1870, –1799, –1220, –334, and –319, respectively. In addition, several phytohormone-responsive elements, light-responsive elements and anaerobic induction elements were also present (Supplementary Table S8). Due to the presence of conserved MYB binding sites, we speculated that the MYB activator LAP1 could bind to the *MtGSTF7* promoter. Next, we performed a Y1H assay to detect the interaction of the LAP1 and *MtGSTF7* promoter, and the positive Y1H assay supported our speculation that LAP1 can directly bind to the *MtGSTF7* promoter *in vitro* (Fig. 7A).

To verify whether the binding of LAP1 could induce the transcriptional activity of the *MtGSTF7* promoter, a dual-luciferase assay was performed. The results showed that LAP1 could markedly induce the expression of firefly luciferase driven by the *MtGSTF7* promoter (Fig. 7B, C), with a LUC/REN (LAP promoter-driven luciferase, compared with Renilla luciferase internal standard) value increased more than 20-fold (Fig. 7D). Taken together, we concluded that LAP1 can bind to the *MtGSTF7* promoter to activate its transcriptional activity.

### MtGSTF7 can rescue anthocyanin, but not the PA deficiency of Arabidopsis *tt19-8* and *tt19-7* mutants

**Fig. 8.**
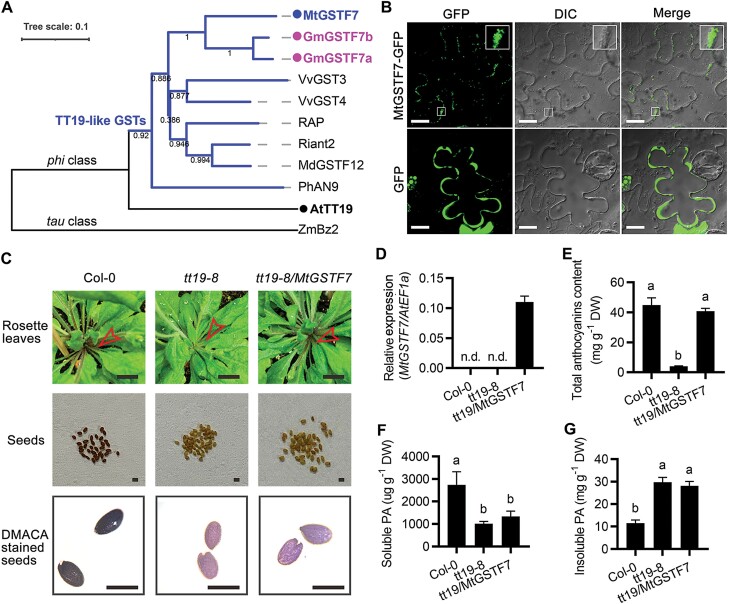
MtGSTF7 is not involved in PA accumulation in *A. thaliana*. (A) Phylogenetic tree of MtGSTF7 proteins and other anthocyanin accumulation related-GSTs. MtGSTF7 is the homolog of AtTT19. Neighbor-Joining tree was constructed by the software MEGA 6.06 using the p-distance amino acid substitution model with 1000 bootstrap repetitions, and displayed using the online tool iTOL. The tree scale for branch lengths denotes genetic distance. Bootstrap values are labelled on the middle of the branch. Blue branches indicate TT19-like GSTs clade. (B) The sub-cellular localization of MtGSTF7 in *N. bethamiania* leaf epidermal cells (upper panels). The sub-cellular location of GFP protein was used as the positive control (lower panels). The MtGSTF7-GFP signal is discontinuous around the cell membrane and the magnified images of the signal in the square areas are presented at the top right corner. Scale bars= 25 µm. (C) Phenotypes of Col-0, *tt19-8*, and a representative rescued *tt19-8*/*MtGSTF7* transgenic line. Hollow red arrow heads indicate the petiole of rosette leaves. Scale bars: upper=1 cm; central=0.5 mm; lower=0.5 mm. (D) Relative transcript levels of *MtGSTF7* in Col-0, *tt19-8*, and the rescued transgenic line. The gene *AtEF1a* was used as an internal control. n.d., not detected. (E) The total anthocyanins content of Col-0, *tt19-8*, and the rescued transgenic line. The anthocyanin content was calculated as C3G (cyanidin-3-*O*-glucoside) equivalents. DW, dry weight. (F) The soluble PA content of Col-0, *tt19-8*, and the rescued transgenic line. The soluble PA content was calculated as epicatechin equivalents. (G) The insoluble PA content of Col-0, *tt19-8*, and the rescued transgenic line. The insoluble PA content was calculated as procyanidin B1 equivalents. Data in (D), (E), (F) and (G) are mean values ±SD (*n*=3). Different letters in (E), (F) and (G) donate statistically significant differences between each other (*P*<0.05, Student’s *t*-test).

In Arabidopsis, AtTT19, a *phi* GST protein, has been considered as a carrier to transport anthocyanins from the cytosol to the tonoplast (Sun *et al.*, 2012). Due to the fact that MtGSTF7 is a typical GST protein, we hypothesized that MtGSTF7 might be a homolog of AtTT19, functioning in anthocyanin transport. Therefore, we analysed its phylogenetic relationship with AtTT19 and other anthocyanin accumulation related-GSTs, based on the entire protein sequences. The amino acid sequence alignment showed that MtGSTF7 presented a high amino acid similarity to other GSTs related to anthocyanin accumulation (Supplementary Fig. S9). Additionally, MtGSTF7 was clustered into the *phi* class clade with a group of TT19-like GSTs in the phylogenetic tree (Fig. 8A). Subsequent protein sub-localization analysis demonstrated that MtGSTF7 protein localized to the cytoplasm (Fig. 8B; Supplementary Fig. S10) similar to other TT19-like GSTs, suggesting that it is a homolog of these proteins that might function in the cytoplasm to facilitate the sequestration of anthocyanins into the vacuole.

In the Arabidopsis *tt19-8* (*SALK_105779*) mutant, both anthocyanin and PA accumulation are defective (Wei *et al.*, 2019). To test whether MtGSTF7 can rescue the phenotypes of *tt19-8,* we ectopically expressed *MtGSTF7* in *tt19-8* under the control of the CaMV 35S promoter. All independent transgenic lines displayed red rosette leaf petioles similar to those of the WT (Fig. 8C, D), and the anthocyanin accumulation defects were completely rescued in the rosette leaves (Fig. 8E).

In the *tt19-8* mutant, the seed coat is pale brown at the ripening stage due to the defect of PA accumulation. After long-term desiccation, the colour turns darker to resemble the WT (Kitamura *et al.*, 2004). In order to harvest the same ripening stage, seeds of Col-0, *tt19-8*, and rescued lines were germinated at the same time and grown under the same conditions. Unlike complementation by overexpression of *AtTT19* (Kitamura *et al.*, 2004), the seed coat colour of all positive transgenic lines was not rescued, with a pale brown coloration at the ripening stage similar to *tt19-8* (Fig. 8C). Both DMACA staining assay and PA quantification confirmed that the PA deficiency was not restored by ectopic expression of *MtGSTF7* (Fig. 8C, F, G).

To further confirm our results, we also attempted to complement another Arabidopsis *tt19* mutant allele, *tt19-7*, and again, only the anthocyanin defect was restored (Supplementary Fig. S11). Taken together, all the results suggested that, unlike AtTT19, MtGSTF7 assists anthocyanin, but not PA accumulation in *A. thaliana*.

### MtGSTF7 is not recruited to participate in PA accumulation in *M. truncatula*

**Fig. 9.**
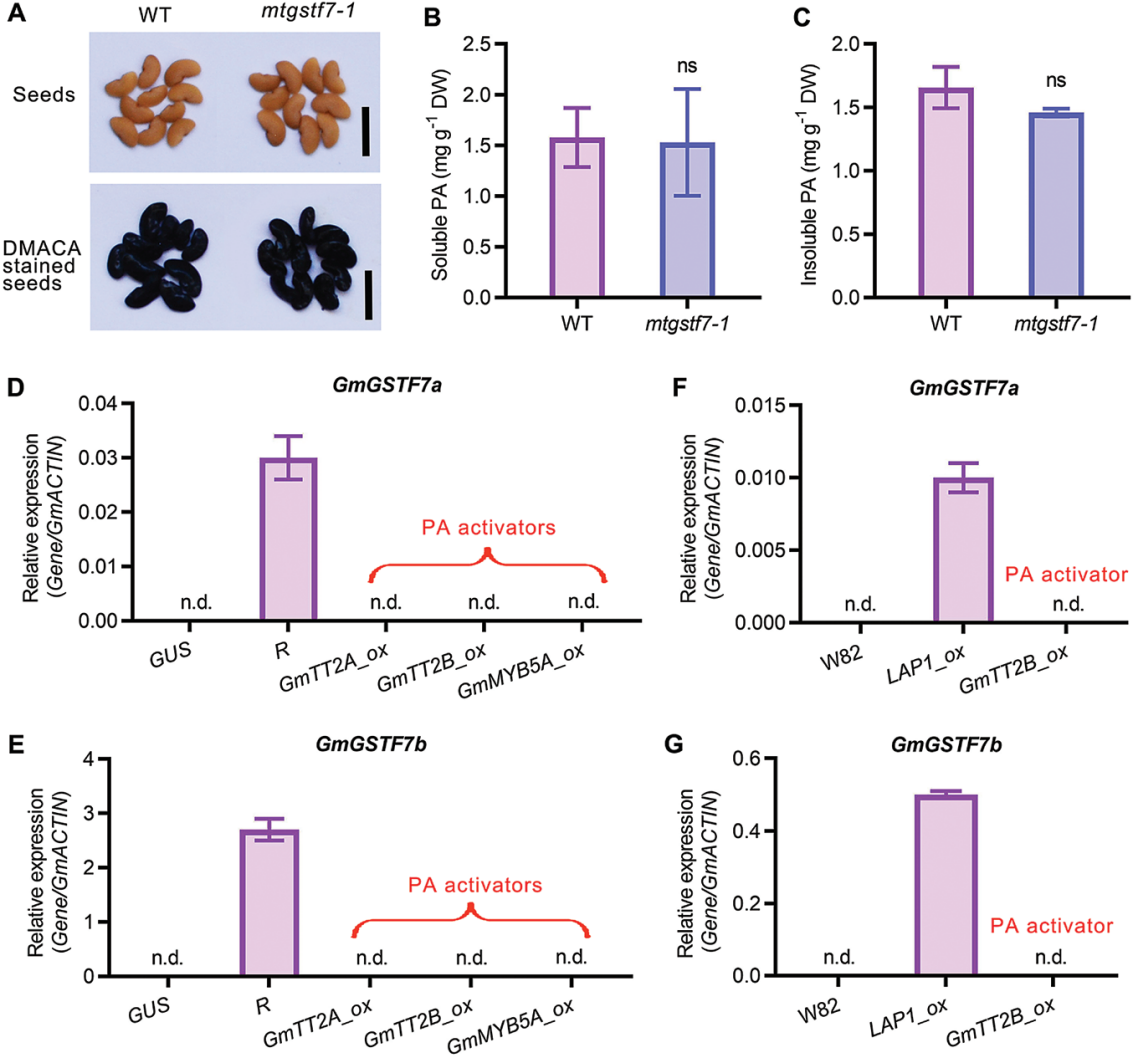
MtGSTF7, and its homologs in soybean, are not responsible for PA accumulation. (A) Phenotype of seeds (upper panel) and DMACA staining of seeds (lower panel) of WT and *mtgstf7-1*. (B) The soluble PA content of WT and *mtgstf7-1* seeds. Scale bars=0.5 cm. (C) The insoluble PA content of WT and *mtgstf7-1* seeds. The soluble PA content was calculated as epicatechin equivalents. The insoluble PA content was calculated as procyanidin B1 equivalents. DW, dry weight. Data in (B) and (C) are mean values ±SD (*n*=4). ‘ns’ above columns denote no statistically significant difference between *mtgstf7-1* and WT (*P*>0.05; unpaired two-tailed Welch’s *t*-test). (D, E) Relative transcript levels of *GmGST7a* and *GmGSTF7b* in *G. max* hairy roots overexpressing *GUS*, *R*, *GmTT2A*, *GmTT2B*, and *GmMYB5A*. Overexpression of *GUS* was used as the negative control. (F, G) Relative transcript levels of *GmGSTF7a* and *GmGSTF7b* in *G. max* (‘Williams 82’) and transgenic plants overexpressing *LAP1* and *GmTT2B.* Gene transcript levels were determined by qRT–PCR. The *GmACTIN* gene was used as the internal control. Data are mean values ±SD (*n*=3). n.d., not detected. GmTT2A, GmTT2B, GmMYB5A are PA activators that induce PA accumulation.

In *M. truncatula*, PAs in the seed coat reach maximal concentration at around 20 d after pollination (Pang *et al.*, 2007). In contrast to the Arabidopsis *tt19* mutants, the seed coat colour of *mtgstf7-1* was similar to that of WT (Fig. 9A). To test whether MtGSTF7 participates in PA accumulation, we stained the WT and *mtgstf7-1* seeds with DMACA reagent. The stained seeds of *mtgstf7-1* showed a similar dark blue colouration to that of WT (Fig. 9A). The quantification results also demonstrated that both soluble and insoluble PA contents were not affected in the *mtgstf7-1* mutant (Fig. 9B, C), indicating that MtGSTF7 is not recruited to participate in PA accumulation in *M. truncatula*.

To verify the homologs of MtGSTF7 in soybean, a phylogenetic tree was constructed to analyse the homology of all *phi* class GSTs in soybean with MtGSTF7 and AtTT19 (Supplementary Fig. S12). The results showed that two homologs of MtGSTF7, namely GmGSTF7a (Glyma.18G043700) and GmGSTF7b (Glyma.11G212900) were present in soybean (Fig. 8A; Supplementary Fig. S12). In our previous study (N. Lu *et al.*, 2021), we observed that *GmGSTF7a* and *GmGSTF7b* showed high expression in transgenic hairy roots overexpressing the anthocyanin master regulator *R* (Supplementary Fig. S13). In contrast, their transcripts were not detected in *GmTT2A*, *GmTT2B* and *GmMYB5A* transgenic lines which accumulated PAs (Supplementary Fig. S13; Supplementary Table S9). A qRT–PCR assay further validated the results (Fig. 9D, E). Besides, the transcript levels of *GmGSTF11a* and *GmGSTF11b* were not detectable in W82 (‘Williams 82’) and transgenic soybean overexpressing *GmTT2B* (Fig. 9F, G), suggesting that these two genes might also play a critical role in anthocyanin accumulation, but are not involved in PA accumulation in soybean.

## Discussion

### MtGSTF7 plays a critical role in anthocyanin accumulation in *M. truncatula*

Anthocyanins are synthesized at the surface of the ER, but require to be sequestered into the vacuole to perform their functions (Grotewold and Davies, 2008). In this study, the mutants of a *phi* class gene, *MtGSTF7*, exhibited completely green plants with no anthocyanin, similar to that of *mtwd40-1* and *mttt8* (Pang *et al.*, 2009; Li *et al.*, 2016). However, the transcript levels of anthocyanin biosynthetic pathway genes in *mtgstf7* were not significantly different from those in WT (Supplementary Fig. S7), suggesting that the green phenotype is the result of a defect in anthocyanin trafficking, rather than a deficiency in anthocyanin biosynthesis. In addition, the ectopic expression of *MtGSTF7* could rescue the anthocyanin deficiency of *tt19* mutants (Fig. 8C; Supplementary Fig. S11), demonstrating that MtGSTF7 plays an analogous function with AtTT19 in terms of facilitating anthocyanin transport from ER to the vacuole.

Previous studies have reported that the tonoplast-located transporter MATE2 showed a preferential transport capacity for anthocyanins (Zhao *et al.*, 2011). Anthocyanin content in *mate2* seedlings is decreased by 2-3 fold compared with WT (Zhao *et al.*, 2011), whereas the accumulation of anthocyanins in *mtgstf7* mutants was almost undetectable. In Arabidopsis, AtTT19 functions prior to the MATE transporter TT12 in PA accumulation (Kitamura *et al.*, 2010). Given that MtGSTF7 is localized in the cytosol (Fig. 8B), it appears that the MATE2-mediated anthocyanin trafficking route might also rely on the function of MtGSTF7. Furthermore, the massive accumulation of anthocyanins induced by overexpressing *LAP1* was notably reduced in *mtgstf7* (Fig. 5A; Supplementary Fig. S8A), indicating that MtGSTF7 functions in the cytosol to allow the accumulation of anthocyanins in *M. truncatula*.

Loss of function of *MtGSTF7* did not affect the expression of anthocyanin biosynthetic genes, even in plants transiently overexpressing *LAP1*(Supplementary Fig. S8B). However, we could not detect any anthocyanins in *mtgstf7* mutants and only detected trace amounts in *mtgstf7-1* transiently overexpressing *LAP1* (Fig. 5E). A possible explanation is that the synthesized anthocyanins in *mtgstf7* mutants might be directly oxidized, degraded or converted to other metabolites in the cytosol. It is reported that the expression of flavanol biosynthetic genes is increased and more flavanols are accumulated in *tt19-7* (Sun *et al.*, 2012). In the *tt19-8 PAP1-D* double mutant line, flavonoids, especially naringenin and quercetin, are significantly increased (Jiang *et al.*, 2020). In the present work, *MtFLS* is more than 50-fold up-regulated in *mtgstf7* compared with WT (Supplementary Fig. S14), indicating the possibility that metabolic flux might be redirected to other flavonoid biosynthetic pathways in *mtgstf7* mutants.

### Light-induced anthocyanin accumulation mediated by MtGSTF7 is independent of LAP1

Several studies have demonstrated that the regulation of anthocyanin accumulation in dicotyledons is conserved, and that the sixth sub-group of MYB TFs plays important roles in anthocyanin accumulation both spatially and temporally (Zhang *et al.*, 2014). In *M. truncatula*, there are 14 members of the sixth sub-group of MYB TFs (Li *et al.*, 2019), indicating that these paralogs might exhibit functional specialization or redundancy in the regulation of anthocyanin biosynthesis. Among those paralogs, apart from LAP1 which was identified by reverse genetics, the other three members, WP1, RH1 and RED HEART2 (RH2), have been demonstrated to participate in tissue specific-accumulation of anthocyanins (Peel *et al.*, 2009; Meng *et al.*, 2019; Wang *et al.*, 2021). WP1 is involved in anthocyanin accumulation in petals (Meng *et al.*, 2019), and RH1 and RH2 precisely regulate the red circle anthocyanin leaf marking on the adaxial side (Wang *et al.*, 2021). So, it is very possible that other members of the sixth sub-group of MYB TFs or other TFs might participate in light-induced anthocyanin accumulation, rather than LAP1, which is of low abundance and is not induced by light (Fig. 6F).

### Other independent anthocyanin transport mechanisms might be present in *M. truncatula* flowers

In addition to GST-mediated anthocyanin transport, vesicle trafficking, ER-to-vacuole protein sorting, and microautophagy-mediated anthocyanin transport across the cytosol, have also been proposed (Poustka *et al.*, 2007; Gomez *et al.*, 2011; Chanoca *et al.*, 2015). Furthermore, it has been reported that homologs of mammalian membrane protein BTL (bilitranslocase) perform anthocyanin transport in carnation petals and grape berries (Braidot *et al*., 2008*a*, *b*). Although anthocyanins were notably absent in vegetative organs, their deposition in flowers was not affected in *mtgstf7-1*, suggesting that the anthocyanin transport mechanisms in vegetative organs and flowers are different, and flowers possibly recruit other functionally redundant GST family members, or use other transport mechanisms. It is possible that anthocyanin biosynthesis is spatiotemporally regulated in *M. truncatula* by different MYB transcription factors, whereby WP1 specifically regulates anthocyanin biosynthesis in flowers (Meng *et al.*, 2019). Furthermore, transient overexpression of *LAP1* still induced trace amounts of anthocyanin accumulation in *mtgstf7*. It is therefore reasonable to assume that, although MtGSTF7 facilitates the majority of anthocyanin trafficking across the cytosol, other mechanisms might also function in parallel in *M. truncatula.*

### MtGSTF7 and its homologs in soybean are not recruited to be involved in PA accumulation

In Arabidopsis, AtTT19 is involved in the accumulation of both anthocyanins and PAs (Kitamura *et al.*, 2004; Sun *et al.*, 2012). Mutants of *AtTT19*, *tt19-7*, and *tt19-8* exhibit anthocyanin and PA-defective phenotypes with green rosette leaves and pale seed coats. In this study, we found that MtGSTF7 could not complement the defective PA phenotypes of *tt19-7* and *tt19-8*. In addition, two null mutants of *MtGSTF7* did not affect PA accumulation in *M. truncatula*. Besides, we did not find *MtGSTF7* transcripts in transgenic hairy roots which massively accumulate PAs (Pang *et al.*, 2008; Verdier *et al.*, 2012; Liu *et al.*, 2014), suggesting that MtGSTF7 is not recruited for involvement in PA accumulation in *M. truncatula*. In addition, its homologs in soybean, *GmGSTF7a* and *GmGSTF7b*, were also not expressed in PA-enriched transgenic soybean or hairy roots. Taken together, it appears that TT19-like GSTs might not be involved in PA accumulation in legumes.

In a previous report, another mutant, *tt19-4*, has been characterized, in which a mis-sense mutation in AtTT19 at amino acid 205 (W205L) influenced PA, but not anthocyanin accumulation (Li *et al.*, 2011). According to the protein sequence alignment of all anthocyanin-related GSTs, W205 is conserved in all TT19-like GSTs, including MtGSTF7 (Supplementary Fig. S9). However, MtGSTF7 still could not rescue the PA deficiency of *tt19*. Therefore, we speculate that, besides W205, other amino acids that are spatially close to W205 might also be required for PA accumulation. Further work is required to address the substrates of TT19-like GSTs, and the functional amino acid residues.

## Supplementary data

The following supplementary data are available at *JXB* online.

Table S1. The mapping file for gene IDs of IMGAG V4.0 versus Affymetrix probe sets IDs.

Table S2. The significant differentially expressed genes (DEGs) in transcriptomic data that was released by Peel *et al.* (2009).

Table S3. The significant DEGs in transcriptomic data that was released by Peel *et al*.

Table S4. The details of 35 common DEGs shared between two transcriptomic datasets reported by Peel *et al.* (2009) and Donna *et al.* (2016).

Table S5. The details of the expression atlas corresponding to five candidate common genes.

Table S6. List of primer sequences used in this study.

Table S7. The chi-square test of the population from *mtgstf7-1* BC_1_F_2_ generation.

Table S8. *Cis*-acting regulatory elements in the *MtGSTF7* promoter.

Table S9. FPKM values of *Glyma.11G212900* and *Glyma.18G043700* in *GmMYB5A* transgenic hairy roots.

Fig. S1. Standard curves of epicatechin and procyanidin B1 used for analysis of PA content.

Fig. S2. Venn diagram of differentially expressed genes (DEGs) from two *LAP1* overexpression transcriptomic datasets.

Fig. S3. The analysis of MtGSTF7 protein sequence.

Fig. S4. The anthocyanin content analysis of WT, *mtgstf7-1* and the representative rescued lines.

Fig. S5. Phenotype of seed pods in WT and *mtgstf7-1* mutant.

Fig. S6. The genetic analysis of the *mtgstf7-1* BC_1_F_2_ population.

Fig. S7. The transcript levels of anthocyanin regulators and biosynthetic genes in WT and *mtgstf7-1* hypocotyls.

Fig. S8. The adaxial side of leaves and the anthocyanin biosynthetic gene transcript levels following transient overexpression of *LAP1*.

Fig. S9. The amino acid sequence alignment of MtGSTF7 proteins and other anthocyanin accumulation-related GSTs.

Fig. S10. The prediction of transmembrane helices in MtGSTF7 protein.

Fig. S11. MtGSTF7 only complements the anthocyanin defective phenotype of *tt19-7*.

Fig. S12. Phylogenetic tree of all *phi* class GST members in *G. max* and *M. truncatula*.

Fig. S13. Expression profiles of *GmGSTF7s* in *G. max* transgenic hairy roots.

Fig. S14. The relative transcript level of *MtFLS* in WT and *mtgstf7-1*.

Fig. S15. Phenotype of flowers in WT and *mtgstf7-1* mutant.

erac112_suppl_Supplementary_Figures_S1-S5_Tables_S6-S9

erac112_suppl_Supplementary_Table_S1

erac112_suppl_Supplementary_Table_S2

erac112_suppl_Supplementary_Table_S3

erac112_suppl_Supplementary_Table_S4

erac112_suppl_Supplementary_Table_S5

## Data Availability

Data supporting the findings of this study are available within the paper and supplementary data published online.
