# Supplementary material for: MtGSTF7, a TT19-like GST gene, is essential for accumulation of anthocyanins, but not proanthocyanins in Medicago truncatula
Source: J Exp Bot. 2022 Mar 16;73(12):4129–46. doi: 10.1093/jxb/erac112 (PMC9232208; doi:10.1093/jxb/erac112)
Supplement: erac112_suppl_Supplementary_Figures_S1-S5_Tables_S6-S9 [file erac112_suppl_supplementary_figures_s1-s5_tables_s6-s9.pdf]

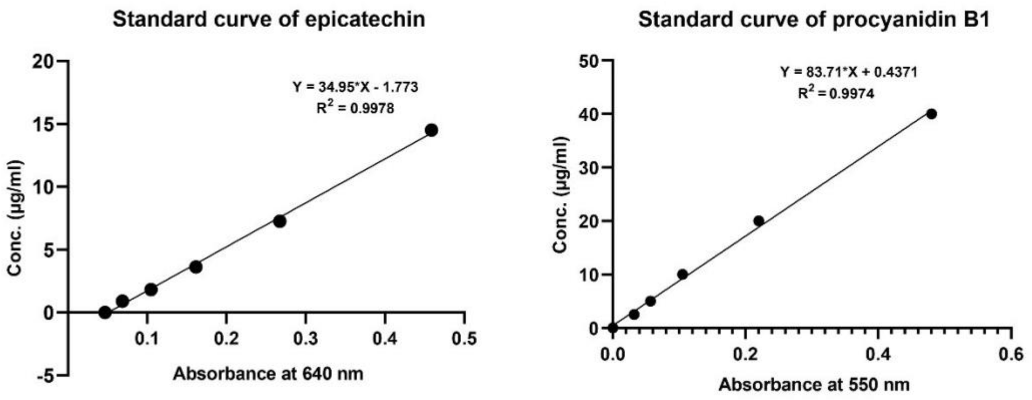

Figure S1. Standard curves of epicatechin and procyanidin B1 used for analysis of PA contents. The standard curve of epicatechin was used to analyze the soluble PA contents. The standard curve of procyanidin B1 was used to analyze the insoluble PA contents.

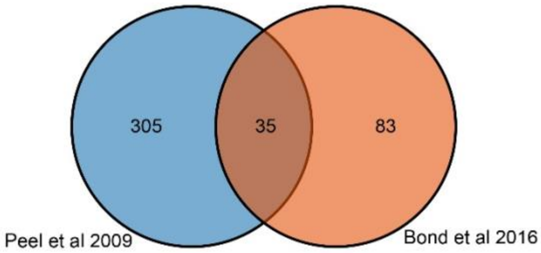

Figure S2. Venn diagram of differently expressed genes from two *LAP1* overexpression transcriptomic datasets. There are 35 common genes were shared between two transcriptome data. The Venn diagram was plotted using the TBtools software.

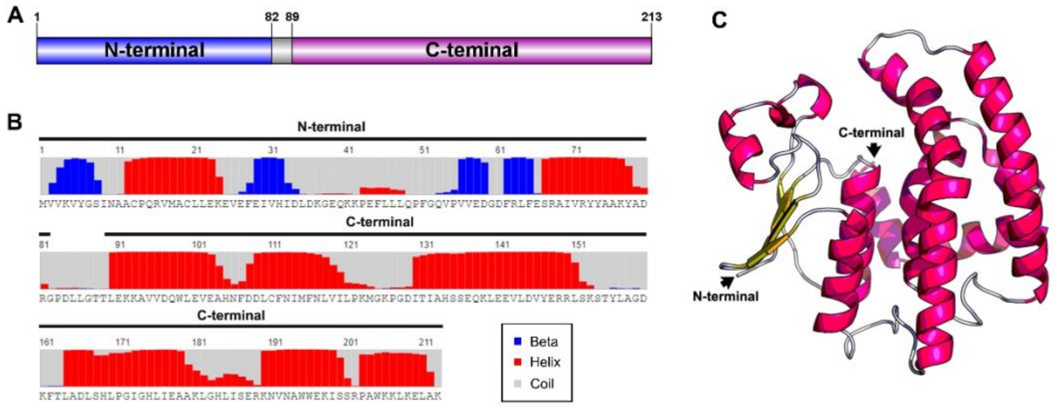

Figure S3. The analysis of MtGSTF7 protein sequence.

(A) Schematic showing the Medtr3g064700 protein. Numbers represent the amino acid numbers. N-terminal and C-terminal show the canonical glutathione-S-transferase N-terminal and C-terminal regions, respectively. (B) The predicted secondary structure of Medtr3g064700 protein in RaptorX. (C) The predicted 3D-structure model of Medtr3g064700 in RaptorX.

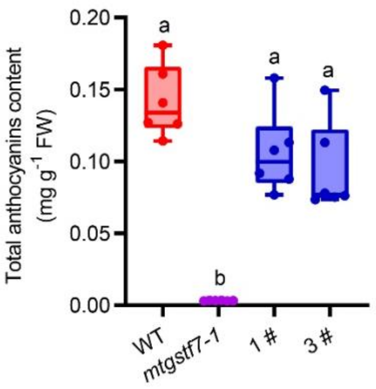

Figure S4. The anthocyanin content analysis of WT, *mtgstf7-1* and the representative rescued lines.

The total anthocyanin contents of hypocotyls from WT, *mtgstf7-1* and the representative rescued lines. The anthocyanin content was calculated as cyanidin chloride equivalents. FW, fresh weight. The data are mean values ± SD (n=6). Different letters denote significant differences between each other ($P < 0.05$; One-way ANOVA test).

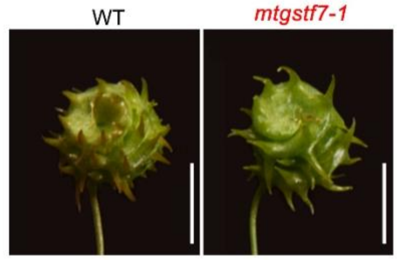

Figure S5. Phenotype of seedpods in WT and *mtgstf7-1* mutant.

The pods of WT and *mtgstf7-1* mutant. The seedpod spines of WT are reddish. WT and mutant plants were grown under the same conditions with high light. Scale bars: 5 mm.

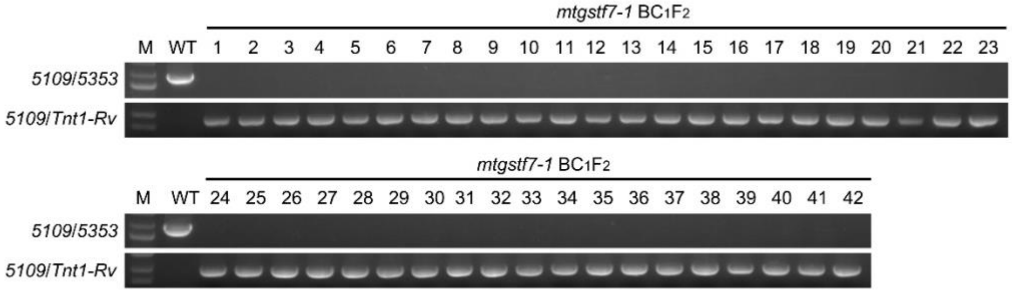

Figure S6. The genetic analysis of the *mtgstf7-1* BC_1F_2 population.

The genotyping of the 42 individual mutants segregated from the BC_1F_2 population. The homozygous *Tnt1* insertion of *MtGSTF7* co-segregated with the 42 BC_1F_2 mutants. Primers *5109* and *5353* are specific forward and reverse primers used for amplifying a fragment of *MtGSTF7* which are shown in Figure 4C.

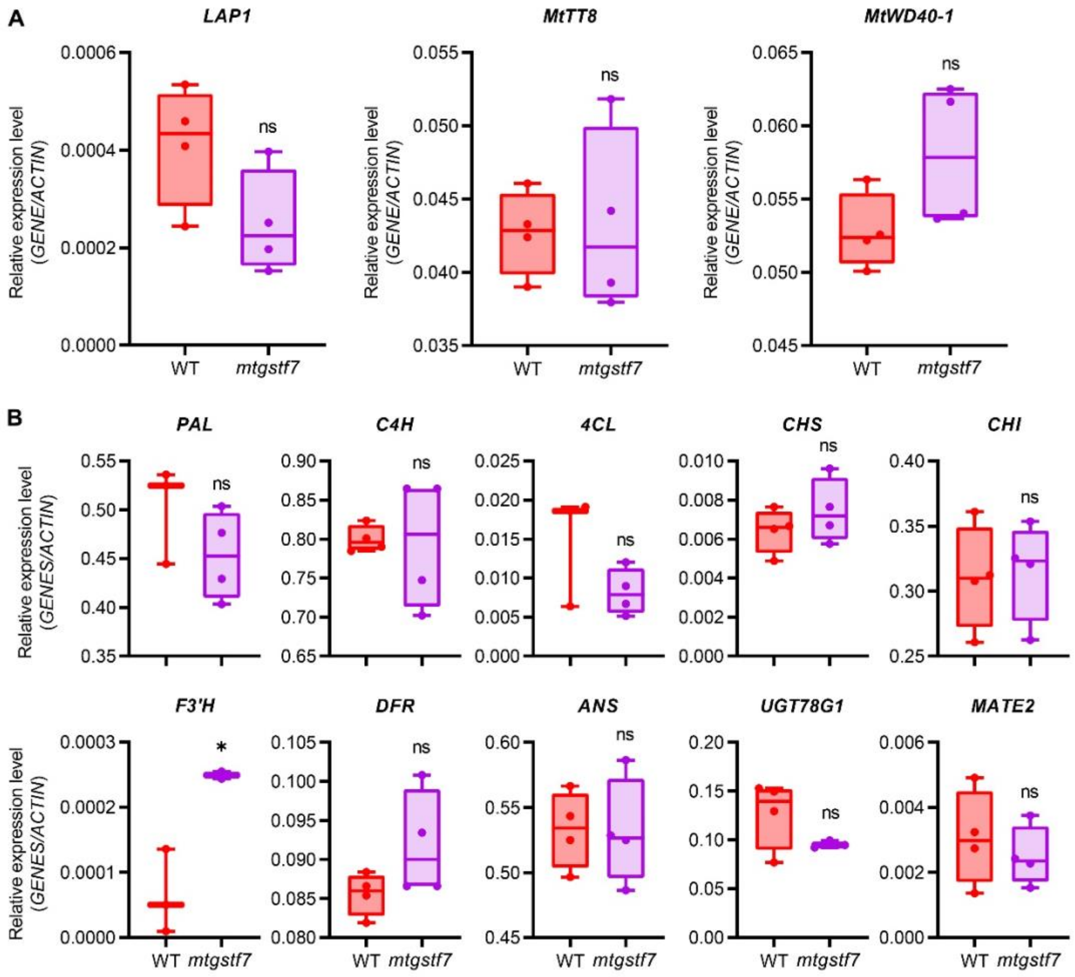

Figure S7. The transcript levels of anthocyanin regulators, biosynthetic genes, and transporter in WT and *mtgstf7-1* hypocotyls.

Transcript expression levels of genes were determined by qRT-PCR. The data are mean values ± SD (ns, $P > 0.05$; *, $P < 0.05$; unpaired two-tailed Welch's t-test).

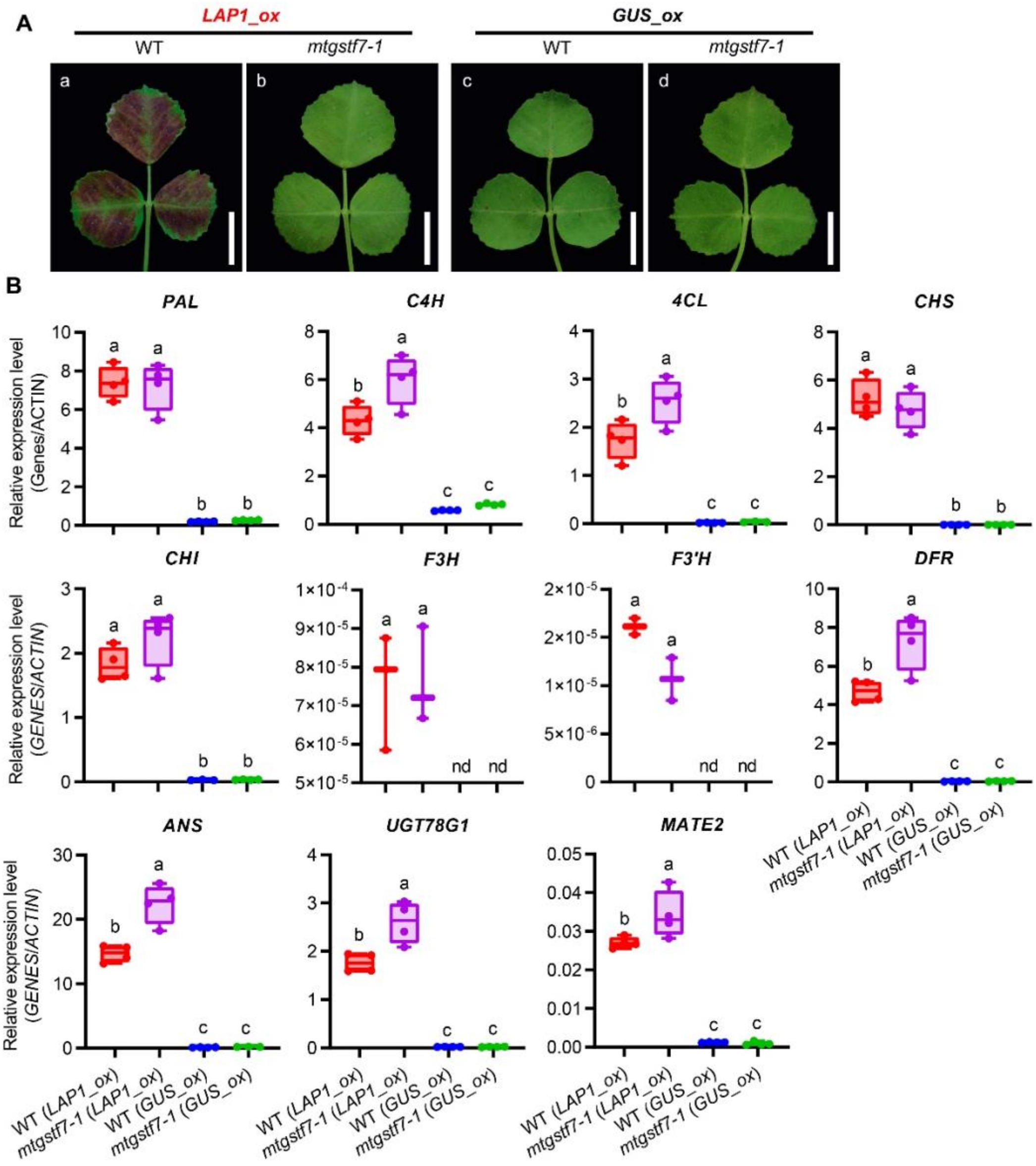

Figure S8. The adaxial side of leaves and the transcript levels of anthocyanin biosynthetic gene and transporter following transient overexpression of *LAP1*.

(A) The adaxial side of leaves that transiently overexpress *LAP1* and *GUS*. Transient overexpression of *GUS* was used as the negative control. Scale bars: 1 cm. (B) Relative expression levels of genes determined by qRT-PCR. The data are mean values ± SD (n=4). Different letters indicate significant differences between each other ($P < 0.05$; Two-way ANOVA tests).

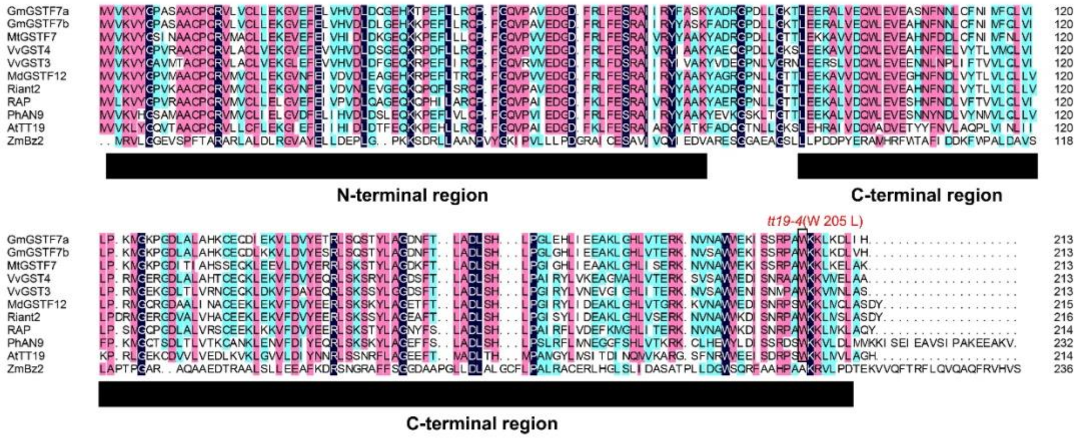

Figure S9. The amino acid sequence alignment of MtGSTF7 proteins and other anthocyanin accumulation related GSTs.

Full length protein sequences were aligned by the DNAMAN software with the default parameters. Conserved N-terminal and C-terminal domains are labeled.

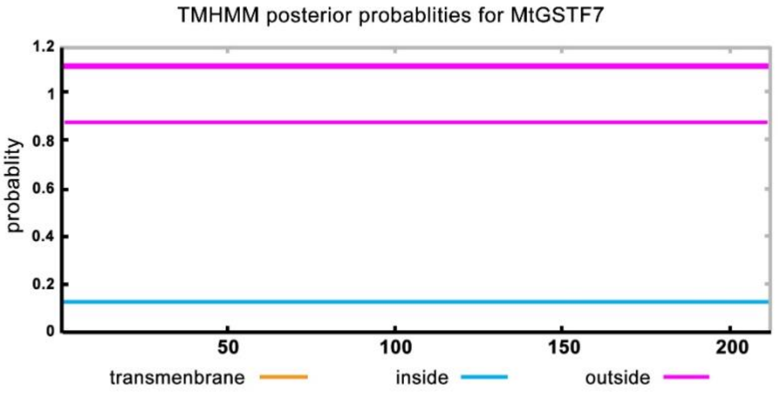

Figure S10. The prediction of transmembrane helices in MtGSTF7 protein. No transmembrane helices in MtGSTF7 were predicted using the Online tool “TMHMM Server v. 2.0”

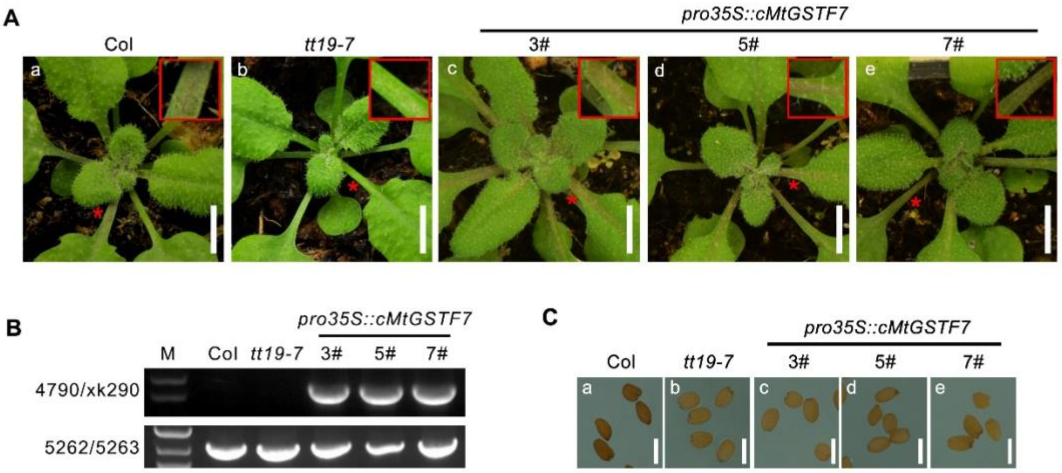

Figure S11. MtGSTF7 only complements the anthocyanin defective phenotype of *tt19-7*.

(A) MtGSTF7 complementing the anthocyanin deficiency phenotypes of Arabidopsis *tt19-7*. Magnified images showing the anthocyanin accumulation in petioles. (B) The genotyping of the three independent complementary transgenic lines. The primer xk290 was the specific reverse primer located at the NOS terminator of the construct. The primer pair of 4790/xk290 was used to identify the positive transgenic lines. The primer pair of 5262/5263 was used as the control to specifically amplify *AtYODA*. (C) The light brown color of the seed coat in *tt19-7* cannot be complemented by MtGST7 overexpression.

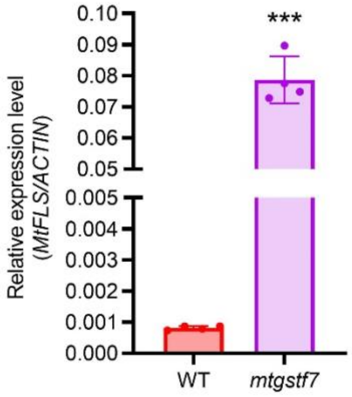

Figure S12. The relative transcript level of *MtFLS* in WT and *mtgstf7-1*.

Data are mean values ± SD (n=4) (***, $P < 0.001$; unpaired two-tailed Welch's t-test).

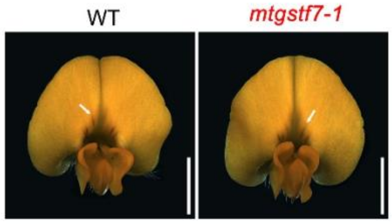

Figure S13. Phenotype of flowers in WT and *mtgstf7-1* mutant

(A) The representative flowers of WT and *mtgstf7-1* mutant. Arrows indicate the dark stripes on the vexillum petals caused by anthocyanin deposition. Scale bars: 2 mm.

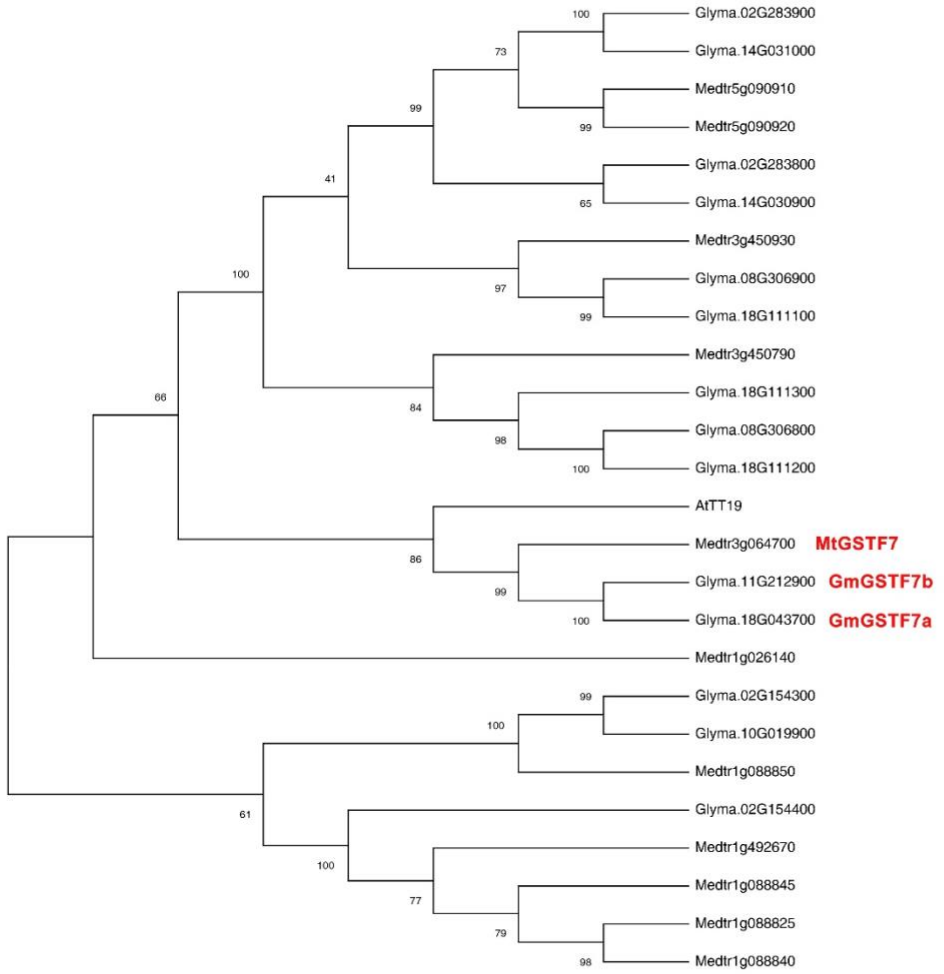

Figure S14. Phylogenetic tree of all *phi* class GST members from *G. max* and *M. truncatula*. Numbers next to nodes represent confidence values.

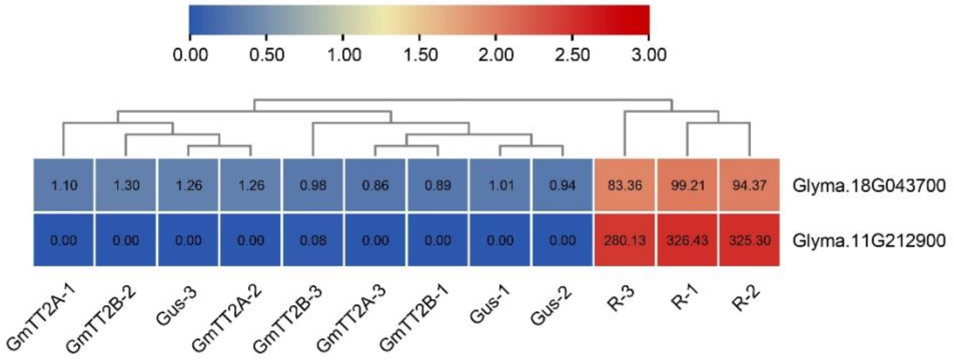

Figure S15. Expression profiles of *GmGSTF7s* in *G. max* transgenic hair roots.

The heatmap was constructed by the TBtools software. Hierarchical clustering adopted Euclidean distance and complete linkage cluster method. Rows were clustered using the Log_{10} based transformed values. The scale legend showing the degree of gene transcript levels. Values in the rectangles showing the original FPKM values acquired from the soybean RNA-seq data (https://onlinelibrary.wiley.com/doi/10.1111/pbi.13562).

Table S6. The chi-square test of the population from *mtgstf7-1* BC_1F_2 generation.

BC_1F_2 Phenotype	Observed value	Expected value	Expected ratio	χ^2	P-value
**WT**	139	135.75			
**Mutant**	42	45.25			
			3:1	0.27071	0.57692

Note: $df = 1$, χ^2 (0.5) = 3.84.

χ^2 denotes the corrected chi-square value; P-value was the asymptotic significance calculated by IBM SPSS20 software.

Table S7. Cis-acting regulatory elements in the *MtGSTF7* promoter.

Name	Position	Sequence	Function
**ABRE**	-1721, -801, -207	AACCCGG/ACGTG	Abscisic acid responsiveness
**CGTCA-motif**	-763,	CGTCA	MeJA-responsiveness
**GT1-motif**	-1404	GGTTAA	light responsive element
**chs-CMA1a**	-406	TTACTTAA	light responsive element
**Box 4**	-1835, -1831, -378	ATTAAT	light responsiveness
**MRE**	-1220	AACCTAA	MYB binding site (light responsiveness)
**MBS**	-1799	CAACTG	MYB binding site (drought-inducibility)
**MYB**	-1902, -1870, -334, -319	TAACCA	MYB binding site
**MYC**	-1326	CATGTG	MYC binding site
**ARE**	-1824, -653, -234	AAAC	Anaerobic induction
**GC-motif**	-198	CCCCCG	Anoxic specific inducibility

Table S8. FPKM values of *Glyma.11G212900* and *Glyma.18G043700* in *GmMYB5A* transgenic hair roots.

Gene	Gus_1	Gus_2	Gus_3	Gus_4	GmMYB5A_1	GmMYB5A_2	GmMYB5A_3	GmMYB5A_4
**Glyma.11G212900**	0	0	0	0	0	0	0	0
**Glyma.18G043700**	0.609185	0.648461	0.773449	0.562921	0.321528	0.40561	0.444581	0.747966

Table S9. List of primer sequences used in this study.

Primer names	Direction	Sequences (5'-3')	Notes of usage
5109	F	CAACCCCCGAGCTGTTAGTT	For genotyping
Tnt1-Rv	R	CAGTGAACGAGCAGAACCTGTG	
5103	F	AAAGTATGCAGACCGTGGT	
5353	R	ACGGTCTGCATACTTTGTTGC	
Xk290	R	cgcgtattaaatgtataattgcgggac	
4790	F	gaacacgggggactcttgacATGGTGGTGAAAGTTTATGGT	For constructing *35S::cMtGSTF7*
4791	R	gggaaattcgagctggtcacTTACTTAGCCAATTCCTTCAAC	
5107	F	agtcgacctgcaggcatgcaTCAAGTTCGTTGCGGTTAGC	For constructing *ProMtGSTF7::gMtGSTF7-3'UTR*
5111	R	gggaaattcgagctggtcacGATTTTATTGTCATATGATG	

5112	F	ttcatttggagaggacacgcATGGTGGTGAAAGTTTATGGT	For constructing *35S::cMtSGTF7-GFP*
6307	R	gttcttctcccttacccatCTTAGCCAATTCCTTCAAC	
6240	F	tacgctcatatgggatccATGGAGAATACCGGAGGTGT	For constructing *AD-LAP1*
6241	R	attattgacacgcccgggTCAAGGTAGATCCCAAAGA	
5893	F	atgatgaattgaaaagcttgaTTTCAGAATACCCCCTAATAG	For constructing *pABAi-ProMtGSTF7*
6366	R	ATGGTGTCCCAGTCTAAGCTTAAAGAGCACTTACA	
6365	F	TGTAAGTGCTCTTTAAGCTTAGACTGGGACACCAT	
5894	R	cagagcacatgcctcgaggtATTTCCCCTGCTCTTTA	
5354	F	gaacacgggggactcttgacATGGAGAATACCGGAGGTGT	For constructing *35S::LAP1*
5355	R	gggaaattcgagctggtcacTCAAGGTAGATCCCAAAGA	
6152	F	AAAACTGCAGATTTTGATGCCTCTACAGAACT	For constructing *proMtGSTF7::LUC*
6375	R	CATGCCATGGATTTCCCCTGCTCTTTA	
MtTUB-RTF	F	TTTGCTCCTCTTACATCCCGTG	MtTUB RT-PCR
MtTUB-RTR	R	CAGCACACATCATGTTTTTGG	
5114	F	CCTTCTCCTCCAGCCCTTTG	*MtGSTF7* RT-PCR
5115	R	GTCCCAATTTTGCTGCCTCA	
2383	F	TCAATGTGCCTGCCATGTATGT	*MtACTIN* qRT-PCR

2384	R	ACTCACACCGTCACCAGAATCC	
2385	F	GACTTTATTGGTGATACCAGGTCG	*MtGAPDH* qRT-PCR
2386	R	GGTCAACCACACGGGTACTGTAA	
2928	F	CTCCAGCCCTTTGGTCAAGTT	*MtGSTF7* qRT-PCR
5353	R	ACGGTCTGCATACTTTGTTGC	
AtEF1aF	F	AGGTCCACCAACCTTGACTG	*AtEF1a* qRT-PCR
AtEF1aR	R	GAGACTCGTGGTGCATCTCA	
AtACTIN2-qRTF	F	TCCCTCAGCACATTCCAGCAGAT	*AtACTIN2* qRT-PCR
AtACTIN2-qRTR	R	AACGATTCCTGGACCTGCCTCATC	
GmGSTF11a-qRTF	F	GAATGGAGGTGTTTAATAATTAAAAAACC	*GmGSTF11a* qRT-PCR
GmGSTF11a-qRTR	R	GCCTTTTATGTTTTTACTTAAATGACACAT	
GmGSTF11b-qRTF	F	GGTGTGTGTCATGTTTTATTATTACG	*GmGSTF11b* qRT-PCR
GmGSTF11b-qRTR	R	TCTTGACCATTCAATAAGCTGCATAA	
GmACTIN-qRTF	F	CTTCCCTCAGCACCTTCCAA	*GmACTIN* qRT-PCR
GmACTIN-qRTR	R	GGTCCAGCTTTCACACTCCAT	
GmCONS4-qRTF	F	GATCAGCAATTATGCACAACG	*GmCONS4* qRT-PCR
GmCONS4-qRTR	R	CCGCCACCATTCAGATTATGT	

9295	F	ACCGACGAAAACGGCAAGAA	*GUS* qRT-PCR
9296	R	CACGGTGATATCGTCCACCC	
5848	F	CCCCAACATCAACAGAGGAAGA	*LAP1* qRT-PCR
5849	R	TTCTTTGCCAAATTTGTGTGCC	
2894	F	GTGGTGTAACGGTTTTGGTGG	*MtTT8* qRT-PCR
2895	R	CGAAACAACCTCCGTCTCAAC	
6075	F	ATTCCCCACAACAACGTATCG	*WD40-1* qRT-PCR
6076	R	ATGCGGTTTGTGTATTCTTCGA	
2874	F	TCTTGGTGGCGAAACACTGAC	*PAL* qRT-PCR
2875	R	TCCATCACCCAATCACTGCTG	
2882	F	TCTACGAATGGCGATCCCACT	*C4H* qRT-PCR
2883	R	TGTTTGCAAGCCACCATGC	
2880	F	CTCTCCGAATTTGCTGATCGG	*4CL* qRT-PCR
2881	R	CAAACCAGCCGCGATTTTTT	
2864	F	CTGCAAACCCAGCCAATTGT	*CHS* qRT-PCR
2865	R	CACACATGCGTTGGAATTTCTC	
5836	F	CACGCTGTTTCCCCTGATCT	*CHI* qRT-PCR

5837	R	TCAACAACGCCGGTAATCTTG	
2876	F	TTGATGGAATCGACGACGCT	*F3H* qRT-PCR
2877	R	GCGGCAAATCAAAGAACCCT	
9357	F	TGGGCAGTGATAGGAACGTG	*F3'H* qRT-PCR
9358	R	GTGTTGATGGATGCAAGCGG	
2870	F	TCATGAGACTTATGGAGCGCG	*DFR* qRT-PCR
2871	R	CCTTTGCACCTGGCAGTTCTA	
2872	F	CCAATTTGCCCTCAACCAGA	*ANS* qRT-PCR
2873	R	AGTTGCAAACCTGGCACCA	
4694	F	GCGAATGCAGTTGCCATAAAC	*UGT78G1* qRT-PCR
4695	R	CATCGGAAACCTTACGCTGTG	
4696	F	AGCGATTGCATTCGATGTCAC	*MtMATE2* qRT-PCR
4697	R	CCAACCAGGACAATCCATTCC	
